# Non-synonymous variation and protein structure of candidate genes associated with selection in farm and wild populations of turbot (*Scophthalmus maximus*)

**DOI:** 10.1038/s41598-023-29826-z

**Published:** 2023-02-21

**Authors:** Øivind Andersen, Juan Andrés Rubiolo, Davide Pirolli, Oscar Aramburu, Marina Pampín, Benedetta Righino, Diego Robledo, Carmen Bouza, Maria Cristina De Rosa, Paulino Martínez

**Affiliations:** 1 Nofima, PO Box 5010, 1430 Ås, Norway; 2 Department of Animal and Aquacultural Sciences, Norwegian University of Life Sciences (NMBU), Ås, Norway; 3 Department of Zoology, Genetics and Physical Anthropology, Faculty of Veterinary, University of Santiago de Compostela, 27002 Lugo, Spain; 4 Institute of Chemical Sciences and Technologies “Giulio Natta” (SCITEC), CNR, 00168 Rome, Italy; 5 The Roslin Institute and Royal (Dick) School of Veterinary Studies, University of Edinburgh, Midlothian, EH25 9RG UK

**Keywords:** Ecology, Evolution, Genetics

## Abstract

Non-synonymous variation (NSV) of protein coding genes represents raw material for selection to improve adaptation to the diverse environmental scenarios in wild and livestock populations. Many aquatic species face variations in temperature, salinity and biological factors throughout their distribution range that is reflected by the presence of allelic clines or local adaptation. The turbot (*Scophthalmus maximus*) is a flatfish of great commercial value with a flourishing aquaculture which has promoted the development of genomic resources. In this study, we developed the first atlas of NSVs in the turbot genome by resequencing 10 individuals from Northeast Atlantic Ocean. More than 50,000 NSVs where detected in the ~ 21,500 coding genes of the turbot genome, and we selected 18 NSVs to be genotyped using a single Mass ARRAY multiplex on 13 wild populations and three turbot farms. We detected signals of divergent selection on several genes related to growth, circadian rhythms, osmoregulation and oxygen binding in the different scenarios evaluated. Furthermore, we explored the impact of NSVs identified on the 3D structure and functional relationship of the correspondent proteins. In summary, our study provides a strategy to identify NSVs in species with consistently annotated and assembled genomes to ascertain their role in adaptation.

## Introduction

Marine fish species are often distributed across a variety of habitats differing in environmental conditions, particularly water temperature, salinity, dissolved oxygen and light intensity, which in turn affect the distribution of infectious pathogens that, along with predation, compromise their viability. These environmental factors strongly influence somatic growth and reproduction, and furthermore, all of them represent energetically costly metabolic activities engaged by fish. Metabolism and evolution are closely connected^1^, and the many genes underlying these processes are targets of natural selection that may lead to adaptive divergence in organisms inhabiting heterogenous environments^2–7^. Knowledge about adaptive genetic variation and its spatial structuring is crucial for the sustainable management of wild fish resources, but also for improving production traits by selective breeding of economical important aquaculture species. Genomic sequencing of an increasing number of fish species is contributing to unravelling the broad genetic variation across genomes through identification of thousands of single nucleotide polymorphic sites (SNPs) and their possible association with various traits both in domestic and wild populations.

Turbot (*Scophthalmus maximus*; Scophthalmidae; Pleuronectiformes) is a flatfish widely distributed throughout the European coast in the Northeast Atlantic Ocean from Morocco to the Arctic Circle, including the Baltic Sea, and in the South across the Mediterranean Sea until the Black Sea^8^. The species experiences a diversity of physicochemical environments across its range with a north–south temperature cline from ≈7 °C up to ≈22 °C and with salinities spanning from ≈35 PSU in the North Atlantic Ocean to ≈2 PSU in the northern Baltic Sea^9^. Whereas the juveniles and adults are relatively sedentary, the pelagic larvae possess high dispersal potential mediated by oceanic currents and enhanced by the high fecundity of the species^10^. Genetic diversity and population structure of turbot has been investigated with microsatellites and SNPs, mostly in the North Atlantic Ocean^11–14^ and to a minor extent in the Southern area^15,16^ and adaptive variation, across its full distribution range, was recently assessed using a set of SNPs covering the whole genome^17^. Four main genetic regions: Baltic, Atlantic, Mediterranean and Black Sea, were identified using neutral variation. Consistent signals of divergent selection attributed to salinity and temperature, and stabilizing selection related to salinity, were detected across the turbot genome, including candidate genes at specific regions^18^. Moreover, the same set of markers was used to analyze genetic differentiation between wild and farm populations, as a baseline to evaluate the impact of restocking and farm escapees in the wild^18^. Besides the notable differentiation detected (wild vs farm F_ST_ ~ 0.060), signals of selection mostly attributed to growth and resistance to pathologies were detected at specific genomic regions including candidate genes.

Whereas wild turbot populations have declined over the last decades mainly due to overfishing, this delicious species has become the main flatfish farmed worldwide due to its high commercial value^19^. Intensive farming during six generations has been accompanied by a fast development of genomic resources to identify quantitative trait loci (QTL) and candidate genes associated with: growth^19–23^, temperature tolerance^24^, adaptation to salinity^25^, sex determination^26,27^ and resistance to various pathogens^28,29^. Furthermore, runs of homozygosity and genetic diversity across the turbot genome were analyzed to check for selective sweeps in farm and wild populations. This information was integrated with previously reported QTL-associated markers, candidate genes and outlier loci related to natural or artificial selection, and a robust framework on selection signatures across the turbot genome was obtained^30^. Furthermore, functional data on resistance to the main industrial pathogens obtained from the main immune organs have been comparatively assessed and integrated with previous signatures of selection across the turbot genome^31^. The broad information gathered in this species, both in wild and farm populations, make it a suitable candidate for assessing the relevance of the different sources of genetic variation on turbot adaptation to different scenarios.

Studying association between polymorphisms within candidate genes and traits of interest or environmental variables in populations or families with different genomic background is a convenient approach to validate their putative adaptive role on natural or domestic selective pressures^23,32^. The significant association can be taken as evidence that the gene is either directly involved in the control of the trait or in linkage disequilibrium with the responsible variant due to its vicinity. If non-synonymous variation is considered, association could eventually lead to the identification of the causative mutation as reported in various vertebrates, including teleost fish. In Atlantic salmon (*Salmo salar*), non-synonymous SNPs in two strong candidate genes coding for the epithelial cadherin and the NEDD-8 activating enzyme 1 (NAE1)^33,34^ have been suggested to be responsible for resistance to infectious pancreatic necrosis virus. Differences in spawning time associated with functionally different protein variants have been documented in Atlantic salmon vestigial-like protein 3 (VGLL3) and in herring (*Clupea harengus*) thyrotropin receptor (TSHR)^35,36^. A hemoglobin polymorphism in turbot was reported to be associated with differences in juvenile growth rates^37,38^ and the underlying amino acid substitution was predicted to influence the stability of the oxygen-binding protein^39^.

Advances in modelling three-dimensional (3D) protein structures together with the progressive enrichment on mutation databases are making feasible to approach the interpretation of non-synonymous variation in terms of protein function^40–42^. This information is essential to understand the evolutionary significance of non-synonymous variation associated with environmental variables^43–46^. In silico approaches for predicting the protein 3D structure directly from the sequence information play a key role in filling the gap between the numerous sequences available and the experimentally solved structures^47,48^. In the absence of sequence similarity with other sequences in the protein structure database (PDB), the modelling strategy can rely on threading and ab initio modelling^49,50^ or deep learning^42,51^ to predict protein structure.

The amount of genomic information on adaptive variation in wild and farmed turbot prompted us to ascertain the putative role of non-synonymous variants (NSV) of candidate genes on selection related to environmental variation in nature or associated with target traits in breeding programs of turbot aquaculture. Specifically, we committed to: (i) call NSV using resequencing data over the recently assembled chromosome-level turbot genome; (ii) filter the most consistent and relevant functional variants among the ~ 21,500 protein coding genes in the turbot genome; (iii) select NSV on candidate genes putatively related to osmoregulation, growth and disease resistance; (iv) identify signals of selection across the whole distribution range of the species and farms; and (v) to validate functional differences of the most consistent variants using 3D structural protein modelling. Our results provide a broad map of NSV across the turbot genome and support the role of several candidate variants on adaptation to osmotic changes or growth in wild and domestic populations.

## Materials and methods

### Calling non-synonymous variation in the turbot genome

DNA from ten adults (five males and five females) of commercial size (1.5 kg) coming from the breeding program of a turbot company were re-sequenced using 150 bp PE reads on an Illumina NovaSeq 6000 System to 20 × coverage and individually aligned against the turbot reference genome (GCA_013347765.1)^27^ to screen for SNP variation. Individuals were previously checked for parentage using a set of 9 microsatellites to choose unrelated individuals^52^ representative of the genetic diversity of the broodstock. The origin of the founders was the NE Atlantic Ocean, and this population has been selectively bred for five generations with the support of the microsatellite tool mentioned above to avoid inbreeding while retaining as much genetic diversity as possible. Quality filtering and removal of residual adaptor sequences was conducted on read pairs using Fastp v.0.20.0^53^; then, filtered reads were mapped with the Burrows-Wheeler aligner v.0.7.8 BWA-MEM algorithm^54^ against the turbot genome and SNPs and indels were called using bcftools v1.^55^, discarding those aligned reads with a mapping quality (MAPQ) < 30 and those SNPs with a Phred quality score < 30. Variants were annotated using SNPeff v5.1^56^ taking as reference the updated turbot chromosome-level genome assembly (GCA_013347765.1^27^).

### Filtering of NSV: reliability and functional information

The thousands of NSV detected were filtered following functional, technical and population genetics criteria to obtain a map of the most consistent NSV across the turbot genome following previous filtering pipelines reported for the species^27,57^. Functional criteria included: (i) dismiss putative pseudogenes using a conservative criterion, to say, those genes with 3 or more NSVs were discarded; (ii) remove non-sense variants producing truncated proteins; and (iii) discard genes with low-quality annotation. Technical criteria included: (i) availability of ± 100 bp without additional variation which could compromise primer annealing and PCR amplification for further genotyping; (ii) compatibility of the adjacent regions selected for designing multiplex primer panels for genotyping; (iii) validation of the in silico detected allelic variants with the MassARRAY technology^58^. Population genetics criteria included: (i) discard SNPs deviated from Hardy–Weinberg proportions (P < 0.01); and (ii) remove tri-allelic SNPs. From this broad NSVs map, we performed additional filtering to focus on the main traits putatively associated with selection in wild or farm populations where previous information was available to choose a final manageable set of SNPs for validation: (i) select the most relevant candidate genes related to growth, osmoregulation and resistance to pathologies crossing previous literature, mostly on fishes, with previous QTL and functional (differentially expressed genes, DEG) data in turbot^28,29,31^; (ii) identifying suggestive genes close to markers associated with signatures of selection (< 500 kb) ^13,14,30^; (iii) discarding deleterious variants from previous information in other species for the same genes available in public repositories (PROVEAN software^59^; (iv) selecting the most diverse SNP per locus (higher MAF: minimum allele frequency). The conservation of the substituted residues in the 18 selected turbot protein variants was examined by blasting against the corresponding proteins in other teleost species available at NCBI (https://www.ncbi.nlm.nih.gov/).

### Population genetics of selected NSVs across the turbot distribution range

#### Sampling

**Figure 1:**
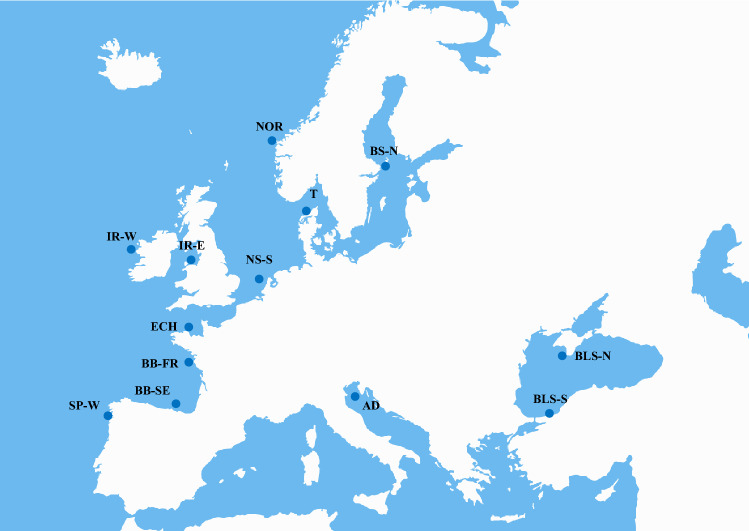
Sampling of wild turbot (*Scophthalmus maximus*) across its European distribution range.

In our screening, we analyzed 13 wild populations including the main genetic regions reported across the turbot distribution range^17^,also representative of the wide variety in temperature and salinity, the main drivers for selection in turbot^17^, but very likely also influencing pathogen distribution^60,61^. We also included samples from the broodstock of the three turbot companies carrying out breeding programs for comparison with wild samples to detect signals of selection related to the main target traits. The broodstock of the three main turbot companies, located in NW Spain and France, were founded with individuals collected from NE Atlantic Ocean^18^, where non-significant genetic differentiation was reported with neutral markers^17^. A total of 355 individuals were analyzed from 16 sampling locations, mostly exceeding 20 individuals/sample (AQUATRACE project; Fig. 1, Table 1). Wild samples included the four main regions of the turbot distribution: Baltic Sea (BAS), Atlantic Ocean (ATL), Mediterranean Sea (MED) and Black Sea (BLS)^17^. The Atlantic Ocean region was overrepresented because of the higher abundance of the species. Farm samples included a representative sample of the broodstock of the three European turbot companies with ongoing breeding programs^18^.

**Table 1:** Sampling information of turbot (*Scophthalmus maximus*). (na, not applicable).

Sample location	Pop code	Sample size	Genetic region	Coordinates (lat-long)
Baltic Sea—North	BAS-N	25	Baltic Sea	60.2/19.7
Skagerak	SK	25	Atlantic	57.4/9.2
Norway Sea	NOR	19	Atlantic	62.0/4.0
North Sea—South	NS-S	24	Atlantic	51.8/2.0
Ireland- West	IR-W	25	Atlantic	59.3/−4.5
Ireland—East	IR-E	25	Atlantic	53.5/−4.8
English Channel	ECH	18	Atlantic	50.7/8.4
Biscay bay—France	BB-FR	23	Atlantic	46.2/−2.2
Biscay bay—Southeast	BB-SE	25	Atlantic	43.4/−3.8
Spain coast – West	SP-W	26	Atlantic	42.6/−8.9
Adriatic Sea	AD	25	Mediterranean	45.2/12.3
Black Sea North	BLS-N	17	Black Sea	44.6/33.4
Black Sea South	BLS-S	28	Black Sea	41.1/31.1
Domestic	Farm 1	14	na	na
Domestic	Farm 2	11	na	na
Domestic	Farm 3	25	na	na

#### SNP genotyping

To genotype and validate in silico allelic variants of the SNPs finally selected we used the MassARRAY technology. Briefly, the protocol consists of a two-step reaction: i) PCR amplification of an amplicon of ~ 150 bp including the selected SNP; and ii) mini-sequencing reaction using an internal primer adjacent to the SNP which extends the primer with a dideoxy nucleotide complementary to the SNP variant^58^. Flanking regions of ± 100 nucleotides of the selected SNPs were obtained from the turbot reference genome (GCA_013347765.1). Design of primer multiplexes and MassARRAY genotyping was done at the UCIM-Universitat de Valencia Genomics Platform.

#### Genetic diversity and differentiation

Mean number of alleles per locus (Na) and expected (H_E_) and observed (H_O_) heterozygosities were estimated to assess genetic diversity per locus. Departure from Hardy–Weinberg equilibrium (HWE) and intrapopulation fixation index (F_IS_) were tested for each locus and population. Global F_ST_ across loci was estimated considering all samples, but also wild sample and farm sample groups separately. Analyses were performed using GENEPOP v4.0^62^.

#### Detection of outlier loci

We followed two different statistical approaches to detect outlier loci showing signals of divergent or balancing selection implemented in BAYESCAN v2.1^63^ and ARLEQUIN v3.5^64^, respectively. Outliers were investigated in: (i) all samples, (ii) wild samples, and (iii) wild vs farm samples; additionally, a hierarchical approach was also explored considering two hierarchical groups (wild *vs* farmed); in all cases we used as background the neutral datasets previously reported for the same comparisons by do Prado et al.^17,18^. The following BAYESCAN parameters were used: 100,000 burn-in length, prior odds of 10 and 20 pilot runs, to identify outliers using a q value < 0.05. The FDIST F_ST_ method implemented in ARLEQUIN was used to investigate loss of heterozygosity after selective sweeps regarding F_ST_. For this program we used the following parameters: 50,000 simulations, 100 demes per group and 20 groups when a hierarchical model was applied. In all ARLEQUIN analyses, outliers were identified considering a *P-*value < 0.01, considering it is prone to a higher number of false positives^65^. The hierarchical scenario could only be implemented with ARLEQUIN, because this option is not available in BAYESCAN.

### Protein 3D structure modelling of non-synonymous variants

To find potential template structures for homology modelling, a specific PSI-BLAST sequence search in the Protein Data Bank (PDB) was performed (https://blast.ncbi.nlm.nih.gov/Blast.cgi)^66^. Identified template structures showed large unresolved regions which encompassed point mutations analyzed in the present study. Two different strategies for modelling were therefore undertaken: I-TASSER^48^ and RoseTTAfold^51^. I-TASSER is a metaserver that automatically employs ten threading algorithms in combination with ab initio modelling to build the tertiary structure of a protein as well as replica-exchange Monte Carlo dynamics simulations for the atomic-level refinement. For comparison an algorithm led by artificial intelligence, RoseTTAFold (https://robetta.bakerlab.org) was also used. The presence of intrinsically disordered regions in the proteins was investigated by the following disorder predictors: PONDR^67^, DISOPRED^68^, IUPRED3^69^ and PrDOS^70^.

Homology modelling was used to generate the 3D model structures of the polymorphic turbot HbαD (see Results) together with the turbot Hbβ1 subunit (AWP17400.1) in the deoxy form (T-state). The structure of deoxyhemoglobin of the Antarctic icefish *Pagothenia bernacchii* (PDB code: 1HBH)^71^ was selected as the most appropriate template to generate the tetramer model (sequence identities of 82.3 and 76% for α and β chains, respectively). Twenty models of each Hb variant were built using MODELLER^72^ as implemented in Biovia Discovery Studio. The model with the lowest MODELLER objective function was selected for analysis.

## Results

### Non-synonymous variants and filtering

**Figure 2:**
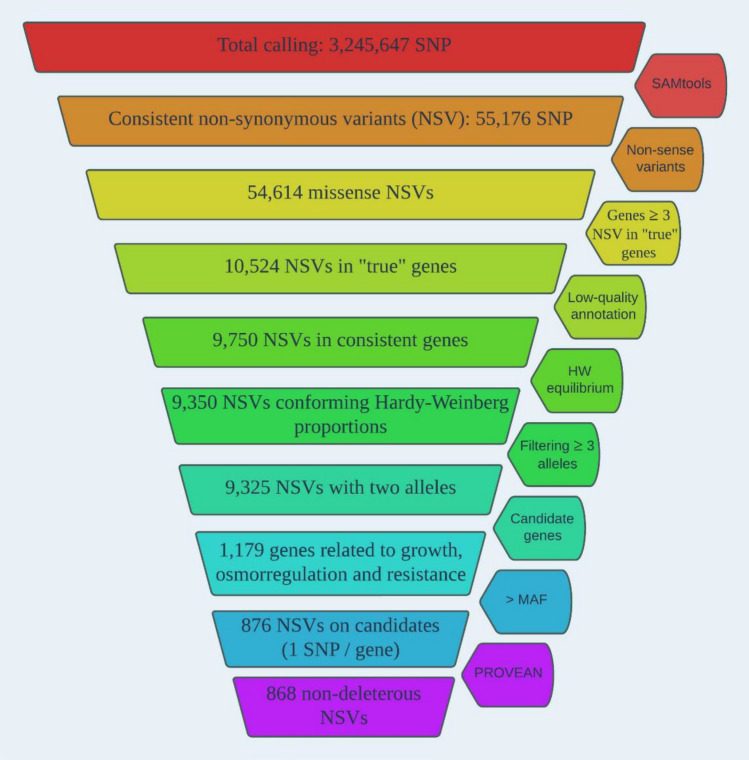
Filtering steps of non-synonymous variants identified in turbot using technical, population genetics and functional criteria.

Among the ~ 3.3 M SNPs detected in the ten 20 × re-sequenced turbot samples, 55,176 represented NSVs after quality control (MAPQ < 30; PHRED < 30; Supplementary Table S1). Among the filtering steps used to select a consistent and manageable set of NSVs for validation, genes with ≥ 3 NSVs (82.9% drop over the previous step) and the functional criterion of selecting genes previously identified associated with growth, osmoregulation and resistance to pathogens (87.4% drop), were the most decisive (Fig. 2; Supplementary Table S1). In the last step, information on candidate genes related to growth and osmoregulation either in turbot or in other fish species (see Introduction for citations), but also for resistance to the main turbot pathogens, *Aeromonas salmonicida* (AS, furunculosis), *Philasterides dicentrarchi* (PD, scuticociliatosis) and *Enteromyxum scohpthalmi* (ES, enteromixosis) was used to retain 1179 SNPs in 876 genes. The SNP with highest MAF for each gene was retained. A total of 84 turbot NSVs were detected in other species using PROVEAN database, and among them, eight were categorized as deleterious and thus discarded for further analyses (Supplementary Table S2). The number of transitions was very similar to that of transversions in the 876 listed NSVs: 432 transitions (A/G = 227; C/T = 205) vs 444 transversions (A/C = 129; AT = 81; C/G = 120; G/T = 115).

### Selection for genotyping and population screening

Our intention was to select a final set of ~ 25 NSVs from the consistent list of 868 candidates to be genotyped in a single multiplex using the MassARRAY technology to validate the reliability of our pipeline and to search for signals of natural or artificial selection in turbot populations across its distribution range. Furthermore, to add functional support, 3D protein structure was evaluated specially on those genes showing significant signals of selection. Accordingly, we focused on genes previously associated with signals of selection in the wild or farm populations related to growth, osmoregulation and resistance to pathogens, detected either by functional assays (DEG: differentially expressed genes) or QTL associations in turbot, but also in other fish species (Table 2). Most of the genes included in the list matched to more than one selection criteria, except for *hbαD* (hemoglobin subunit alpha-D) of particular interest regarding metabolism and growth^39^. The final list included 22 genes associated with growth (13 genes); resistance to ES (13), AS (7) and PD (9); osmoregulation (3); and signals of natural (7) or artificial (3) divergent selection, mostly in turbot, but also from other fish species (10) (Table 2).

**Table 2:** Selection of non-synonymous variants in turbot (*Scophthalmus maximus*) candidate genes following functional criteria.

Gene name	Chrom	Start	REF	ALT	aa substitution	Selection criteria
*hamp*	1	14423573	A	T	N81Y	ES, AS, PD
*fga-like*	2	1113231	G	T	R537Q; A574S	Sma-E137 (GR, OUT) gene; 13831_88 (OUT farm) < 500 kb; PD; AS
*arhgap42*	3	7539292	C	A	T632K; T793N	1916_69 (OUT) gene; GR-OST
*vtna*	3	15402438	G	T	D185E	Sma-USC214 (GR, PD) < 500 kb; PD
*paxbp1*	4	10752137	G	A	P47L	ES; GR-OFS
*cmtm3*	5	10463315	T	C	K83R	5986_20 (OUT farm) < 500 kb; ES
*igf1rb*	5	22614990	A	G	Y980H	Sma-USC7 (GR) gene; GR -OFS
*hmox*	8	13184347	A	G	T81A	7560_71 (OUT farm) and 7235_80 (OUT farm) < 500 kb; PD; AS; ES
*ciart*	10	12731534	A	G	N271S	SmaUSC-E29 (OUT) gene; AS; ES; GR-OFS
*slc12a3*	10	24208615	G	C	D38N; C938S	ES; OR-OFS
*frs2*	10	25869534	G	A	A124T; A290P	SmaUSC-E7 (GR, OUT) gene;
*igfbp2*	14	4200650	G	A	P264S	PD; GR-OFS;
*ccnb1*	16	2499469	C	T	A390V	Sma-USC146 (OUT) < 500 kb; ES
*fgfr3*	17	2378412	G	C	P45R	Sma-USC30 (GR, PD) < 500 kb; ES
*hbαD*	18	9550815	G	A	A44T; V78I	GR-OFS
*aqp8b*	19	11066153	G	T	Q36H	AS, ES; OR-OFS;
*hgs*	19	13543170	C	A	P726T	SmaSNP_298 (GR, PD) < 500 kb; ES
*sstr3*	19	19851767	C	T	S414L	SmaSNP_192 (GR) < 500 kb, ES; GR-OFS;
*tshr*	20	5830531	T	A	L339Q	Sma-USC273 (GR, PD) < 500 kb; AS
*myb*	20	10176750	C	T	C4Y	Sma-USC38 (OUT, PD) < 500 kb; AS
*vipr1b*	21	2341297	T	C	N2D	Sma-E112 (OUT) < 500 kb; Sma-USC91 (GR; VHSV) < 500 kb; ES
*eya3*	22	5576482	G	T	R22L; S230G	ES; GR-OFS;

REF/ALT: reference and alternative alleles; selection criteria: differentially expressed genes for resistance to *E*. *scophthalmi* (ES), *A*. *salmonicida* (AS) and *P*. *dicentrarchi* (PD); genetic markers associated with QTL for growth (GR) or resistance to the same pathogens (ES, AS, PD), and to outliers (OUT) for natural or artificial (farm) selection within the gene (gene) or at less than 500 kb from the gene (< 500 kb); other fish-studies (OST) including osmoregulation (OR) and growth (GR).

### Multiplex design and genotyping on a MassARRAY platform

Among the 22 preselected SNPs, 18 could be included in a single multiplex for MassARRAY genotyping using primers designed from the ± 100 bp flanking regions retrieved from the turbot genome (Supplementary Table S3 and S4). In all cases, the allelic variants detected with MassARRAY genotyping matched with the in silico SNP calling from the re-sequencing turbot data and thus they were validated for further research. Genotypes for the 355 individuals from wild and farm origin were very consistent and only one missing data was detected among the 6390 genotypes (Supplementary Table S5).

### Genetic diversity and differentiation across loci, populations and groups

Global genetic diversity in the wild for the set of 18 SNPs was significantly higher than previously reported using an anonymous SNP panel across the whole genome (Na: 1.77 vs 1.49; H_E_: 0.223 vs 0.090, respectively^17^), which can be explained by the filtering criterion followed for detecting NSVs in this study (at least two variants in the 10 individuals analyzed (20 alleles per locus); minimum allele frequency (MAF) = 0.1). Also, genetic diversity was higher on average in farm than in wild samples (H_E_ = 0.261 *vs* 0.214) even for the Atlantic region (H_E_ = 0.227) suggesting a good management of genetic diversity after five generations of selection. Average genetic diversity per locus ranged from *aqp8b* (aquaporin 8b) (Na = 1.19; H_E_ = 0.0199) to *vipr1b* (vasoactive intestinal peptide receptor 1b) (Na = 2; H_E_ = 0.4664), but other loci, such as *eya3* (eyes absent 3), *hamp* (hepcidin antimicrobial peptide), *fga-like* (fibrinogen-alpha chain-like), *ciart* (circadian-associated transcription repressor) and *tshr* (thyroid stimulating hormone receptor), also showed high genetic diversity figures (Table 3). The remaining loci were polymorphic in most populations (MAF > 0.01). No deviation from Hardy–Weinberg proportions were detected either per locus across populations or per population across loci, excluding Skagerak (SK), which showed a significant excess of heterozygotes for most of the polymorphic loci analyzed (*P* < 0.0023). Interestingly, this population is located in the transition between Baltic Sea and North Sea, where a contact between two highly divergent salinity environments occurs, depicting a rather complex hybridization area^12^.

**Table 3:** Genetic diversity and differentiation of non-synonymous allelic variants in wild and farm turbot (*Scophthalmus maximus*) populations.

Gene	Na	H_E_	F_ST_ (all)	*P*-value	F_ST_ (wild)	*P*-value	F_ST_(farm vs wild)	*P*-value
*aqp8b*	1.19	0.0199	0.0273	0.1223	−0.0019	0.3827	***0.1090***	0.0281
*cmtm3*	1.94	0.1667	0.0065	0.0583	0.0013	0.0505	0.0025	0.0561
*eya3*	2	0.4259	***0.1124***	0.0124	***0.1105***	0.0136	***0.1501***	0.0239
*hamp*	2	0.3944	0.0306	0.2804	0.0274	0.2836	0.0327	0.3380
*hgs*	1.63	0.0439	−0.0094	0.1538	−0.0072	0.1980	−0.0157	0.0859
*igf1rb*	1.75	0.1352	0.0467	0.4232	0.0338	0.4606	0.1057	0.1005
*fga-like*	2	0.4238	0.0690*	0.1809	0.0741	0.1228	0.0943	0.1086
*arhbap42*	1.75	0.0764	0.0065	0.1038	0.0045	0.1143	0.0175	0.2533
*hmox*	2	0.2272	0.0249	0.2195	0.0246	0.2735	0.0170	0.1605
*ciart*	2	0.4572	0.0656	0.2193	0.0539	0.3240	0.0937	0.1270
*igfbp2*	1.19	0.0248	***0.0988***	0.0169	−0.0019	0.3827	**0.3184**	0.0047
*myb*	1.69	0.0667	0.0124	0.1580	0.0157	0.2383	0.0061	0.1268
*paxbp1*	2	0.3464	−***0.0061***	0.0111	−**0.0091**	0.0099	0.0177	0.1639
*slc12a3*	2	0.2656	0.0621	0.2561	0.0623	0.2125	0.0535	0.3941
*sstr3*	1.06	0.0025	−0.0048	0.3020	−0.0019	0.3827	−0.0116	0.1674
*tshr*	2	0.4004	**0.1399**	0.0015	**0.1386**	0.0016	***0.1376***	0.0310
*vipr1b*	2	0.4664	0.0233	0.1831	0.0220	0.2015	0.0146	0.1326
*hbαD*	1.63	0.0715	***0.1399***	0.0249	−0.0084	0.1423	**0.3821**	0.0042

In bold are shown outlier loci due to divergent (high F_ST_) or stabilizing (low F_ST_) selection with ARLEQUIN at P < 0.01 (consistent) or P < 0.05 (italics, suggestive) with respect to neutrality, and with BAYESCAN (p < 0.05, highlighted with *).

**Figure 3:**
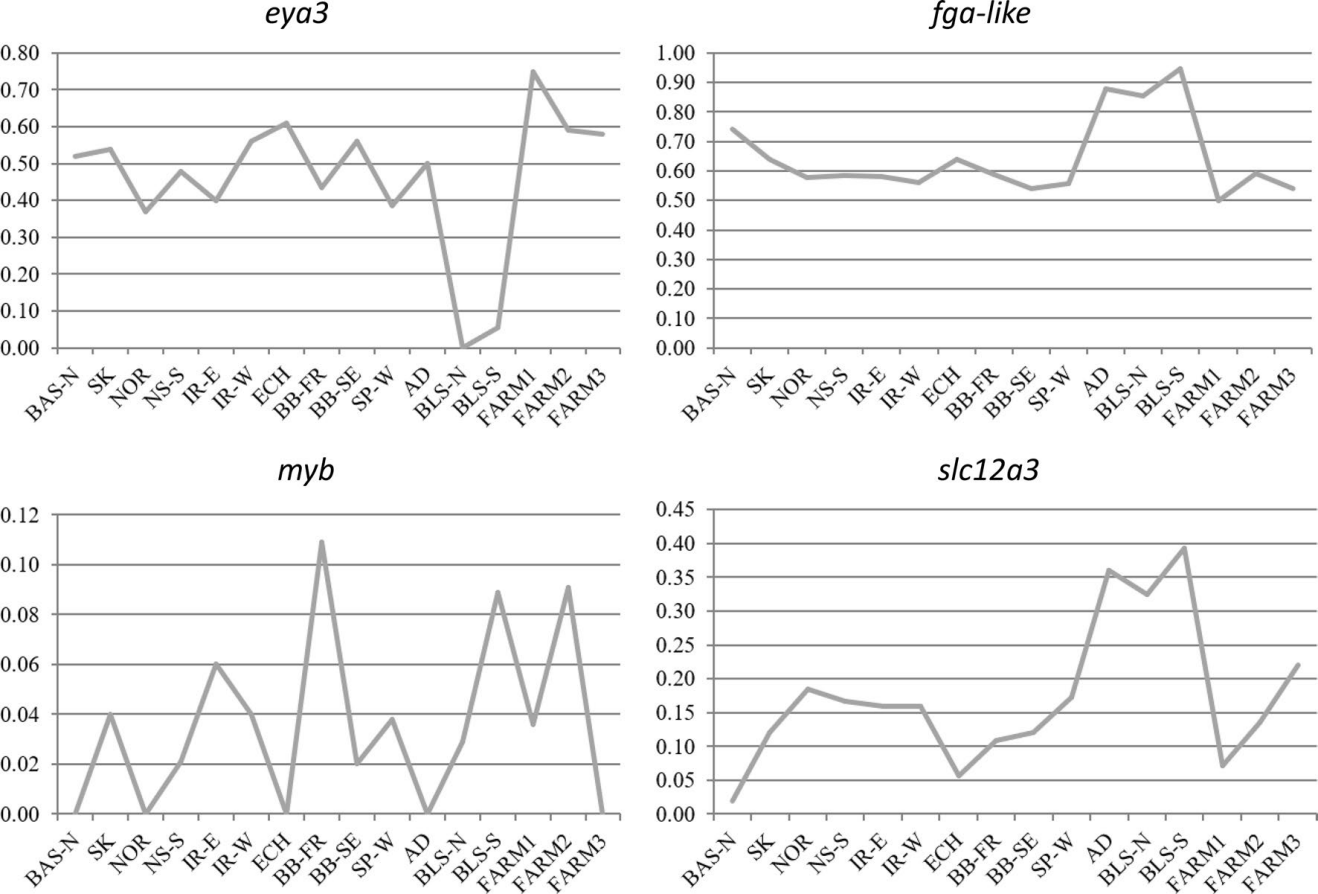
Allele frequency variation for some representative loci including non-synonymous variants evaluated across the whole distribution range and the main farm broodstock of turbot (*Scophthalmus maximus*).

Seven loci showed MAF < 0.1, among which *aqp8b* and *sstr3* (somatostatin receptor 3) showed rare allelic variants (MAF < 0.01). In fact, the *sstr3* locus was nearly fixed for one allelic variant across most populations, while the *igfbp2* (insulin-like growth factor binding protein 2b) and *aqp8* loci were polymorphic at MAF > 0.1 only in one population (Supplementary Table S5). At the other end, *eya3*, *vipr1b* (vasoactive intestinal peptide receptor 1b), *fga-like* and *ciart* were highly polymorphic (MAF > 0.3). Abrupt changes in allele frequencies at some genetic regions or related to the origin of samples (farm, wild) were observed. For instance, it was remarkable the polymorphism decay in the Black Sea of *eya3*, or the increasing/decreasing polymorphism in the southern populations for *slc12a3* (solute carrier family 12 member 3) and *hmox* (heme oxygenase), respectively (Fig. 3). Also, saw peaks showing the effects of genetic drift or sampling variance were observed in the least polymorphic loci, such as *igf1rb* (insulin-like growth factor 1b receptor), *myb* (v-myb avian myeloblastosis viral oncogene homolog) and *cmtm3* (CKLF-like MARVEL transmembrane domain containing 3). Finally, striking variation was also displayed when comparing farm samples between them or to the wild ones.

### Genetic differentiation and signals of selection

We searched for signals of selection on the selected set of NSVs under three different scenarios: i) the 13 WILD populations; ii) ALL the 16 populations (13 wild and 3 farm); iii) comparing wild vs farm populations using a hierarchical approach (HIER). In all cases the set of neutral loci reported by do Prado et al.^17^, when analyzing wild populations, and by do Prado et al.^18^, when comparing wild vs farm populations, were used as the neutral background. A single locus, *fga-like*, which showed a significant decrease of genetic variation in the southern populations, was significant with BAYESCAN (Supplementary Fig. S1), but not with ARLEQUIN. Using the latter software, two loci showed signals of divergent selection, either consistent or suggestive (*P* < 0.01 and 0.05, respectively), in the three comparisons performed: *eya3* was nearly monomorphic in the Black Sea while at intermediate frequencies in the remaining populations; and *tshr* showed a progressive decrease in genetic diversity from the Baltic to the Black Sea, with an abrupt change in the Adriatic Sea, the only Mediterranean population studied. Another locus, *paxbp1* (PAX3 and PAX7 binding protein 1), showed signals of stabilizing selection in two scenarios (WILD, ALL), and close to significance in the third one (HIER). The comparison of wild and farm populations (HIER, ALL) unveiled significant signals of divergent selection for *igfbp2*, *aqp8b* and *hbαD*, which showed a rather similar pattern of differentiation, being monomorphic in nearly all wild populations while the alternative allele increased in two of the farms analyzed. Finally, locus *igf1rb*, although not significant, showed a notable differentiation (F_ST_ = 0.1057) between wild and farm samples (HIER), reaching the highest frequencies of the alternative allele in the same two farms as *igfbp2*, *aqp8b* and *hbαD*.

### 3D structural analysis of the non-synonymous variants with signals of selection

**Figure 4:**
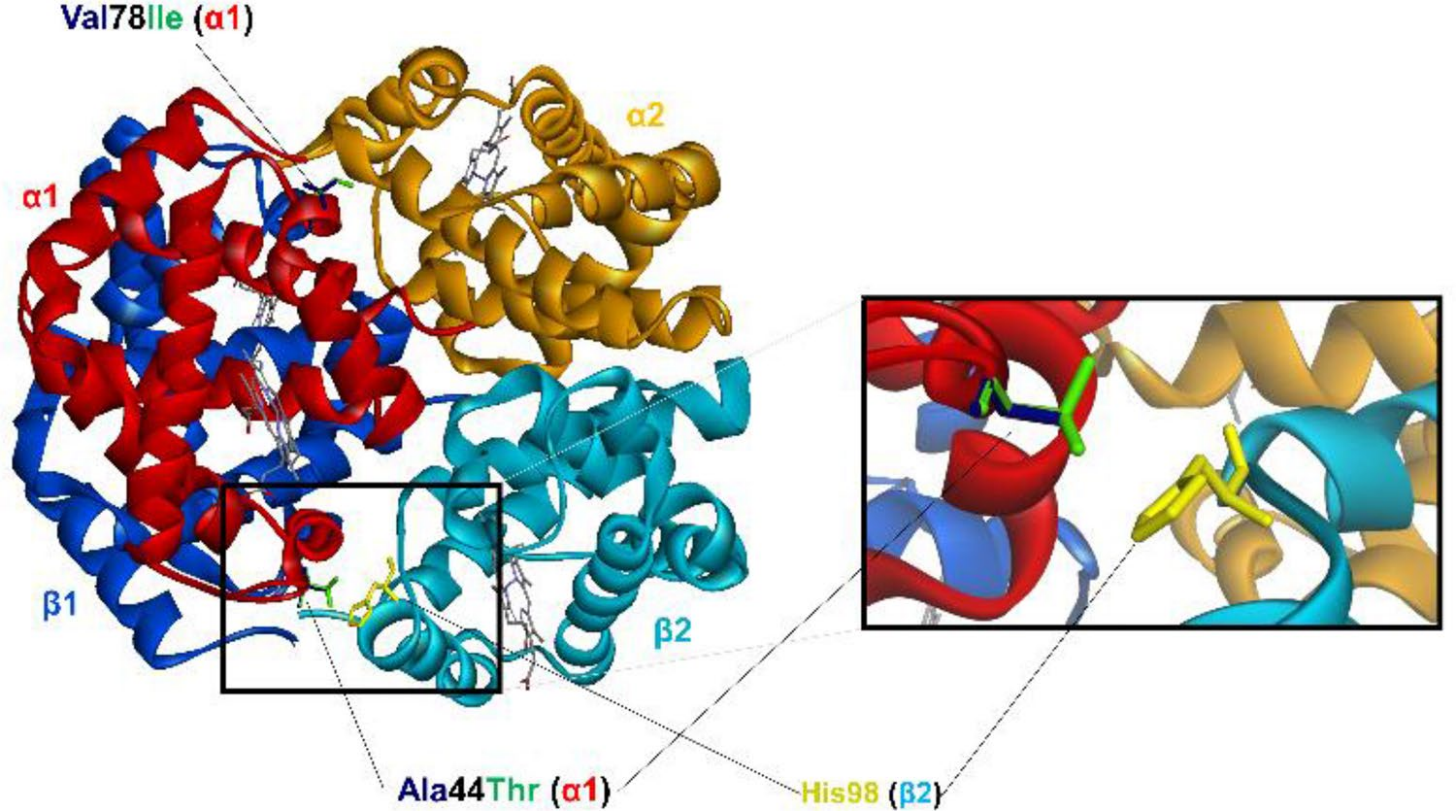
Ribbon representation of the superimposed Ala44Thr and Val78Ile variants of turbot (*Scophthalmus maximus*) HbαD together with the Hbβ1 subunit. Amino acid residues at positions 44α and 78α and the interacting His98β residue are shown in stick. The hydrophobic interaction between Ala44α1 and His98β2 is shown in the enlarged section. The heme groups are shown color-coded by atom type.

The tetrameric deoxyhemoglobin structure of the polymorphic turbot HbαD and Hbβ1 subunits revealed that the Ala44αThr replacement occurs at the α_1_β_2_ interface, which is involved in the allosteric transition of the protein (Fig. 4). The Ala44α variant shows a hydrophobic interaction with His98β that may stabilize the Hb tetramer and so determine a lower oxygen affinity, whereas the interaction is lost upon replacement of Ala with Thr. The conservative Val78αIle substitution does not affect the protein interfaces.

**Figure 5:**
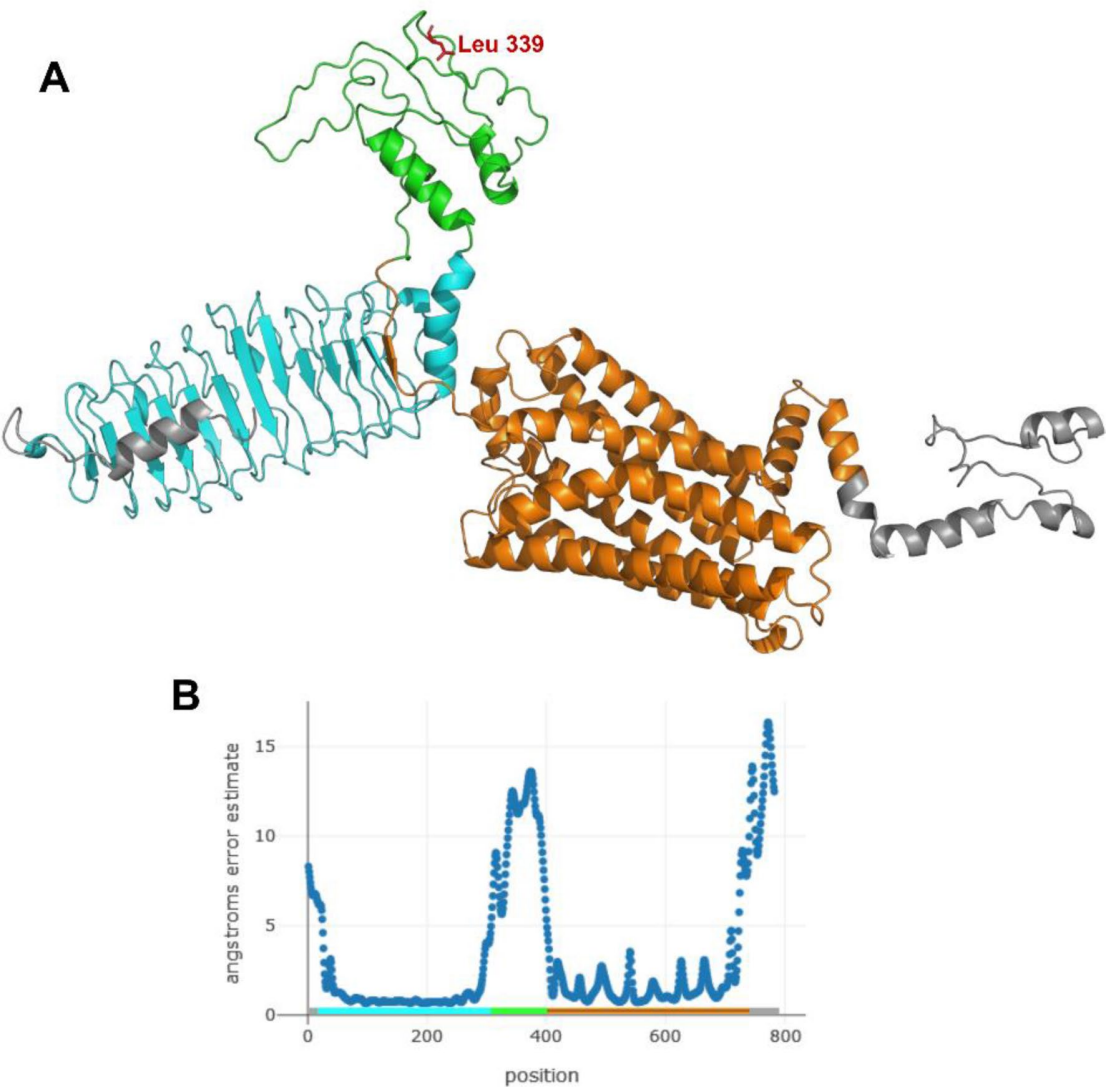
Structural model of turbot (*Scophthalmus maximus*) TSHR. (**A**) Cartoon representation of the hinge region (green) between the seven-parallel-helices domain (orange) and the leucine-reach-repeat domain (light blue). (**B**) Local quality of the model expressed in per-residue error estimate.

**Figure 6:**
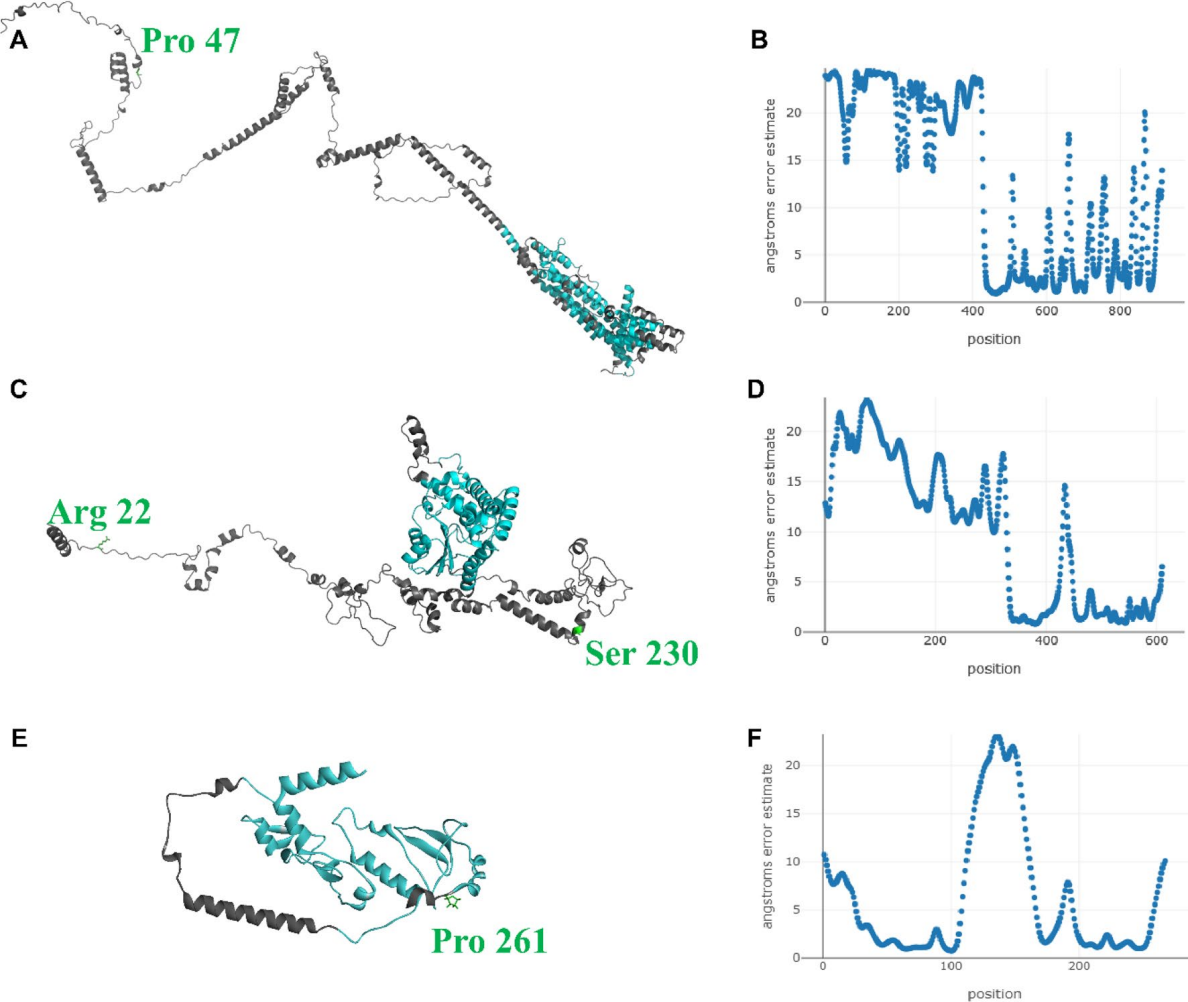
Structural models of turbot (*Scophthalmus maximus*) PAXBP1 (**A**), EYA3 (**C**) and IGFBP2 (**E**) as calculated by RoseTTAfold. Cartoon representation colored by local model quality: low-quality and high-quality in grey and light blue, respectively. The local quality of the three models, expressed in per-residue error estimate, is shown in panel (**B**) (PAXBP1), (**D**) (EYA3) and (**F**) (IGFBP2).

The absence of a suitable template in the PDB for homology modelling of TSHR, PAXBP1, EYA3 and IGFBP2 led us to generate 3D structures using I-TASSER and RoseTTAfold (Supplementary Table S6). The RoseTTAfold TSHR model showed a confidence score of 0.6 and the per-residue error estimate suggests that the Leu339Glu substitution is positioned in an unstructured region from position 298 to 404 (Fig. 5), corresponding to the hinge between the extracellular leucine-rich repeats and the seven-helix transmembrane domain. RoseTTAfold models of PAXBP1 and EYA3 were of low confidence (0.39 and 0.42 scores, respectively), while the IGFBP2 model showed a good confidence score of 0.66, but the C-terminal region containing the Pro261Ser mutation was of low-quality. Modeled structures and corresponding per-residue error estimate are shown in Fig. 6.

## Discussion

Non-synonymous variation plays an important role in evolution and local adaptation to the diverse environment experienced by species with broad distribution ranges^73,74^ and has been profusely screened in humans, *Drosophila* and other model species^75–78^. The increasing genomic resources due to the lowering sequencing costs make it feasible to catch in a quick and cheap way a picture of existing NSVs to be further used to investigate its adaptive role^79–81^. Other sources of variation such as structural variants have been associated with adaptation of fish species in the wild^82^, even in flatfish^83,84^, but the relative importance of NSV and structural on adaptation is still a matter of debate and further studies are needed^85^.

Here, we report the first genome-wide collection of NSVs in the turbot, a flatfish species distributed all around the European coasts, where it experiences gradual and abrupt changes in temperature and salinity^17^. Our study is based on genome resequencing of 10 farm fishes (5 males and 5 females) originated after five generations of selection from breeders of Northeast Atlantic Ocean, the most important region of turbot distribution, thus representing a preliminary picture of NSV in the genome of the species. However, it should be noted that expected heterozygosity was higher in farm than in wild samples, even from the Atlantic region, which shows a good management of genetic diversity in the breeding program. Since 10 diploid genomes were sequenced, our capacity to disclose low frequent and rare variants is limited, especially because filtering included a step for reliability related to MAF = 0.1 (at least two variants in the sample). Nonetheless, we could identify more than 50,000 NSVs across the ~ 21,500 protein coding genes annotated in the turbot genome, which will likely increase when a broader sample including the four main genetic regions identified across its distribution range^17^ is explored. However, since most genetic diversity in the turbot is contained within populations (global NE Atlantic F_ST_ = 0.002^ ns^; global distribution F_ST_ ~ 0.090^17^), our small sample from Northeast Atlantic Ocean would include a significant representation of NSV of the species. After removing those NSVs from putative pseudogenes and those representing non-sense mutations, a set of ~ 10,000 NSV was retained constituting the most reliable set in our study. Considering the expected frequency of NSVs in our small sample (MAF = 0.1) and the high turbot effective population size (usually Ne > 10,000 in the Atlantic Ocean region^17^), it could be assumed that most of this variation is not strongly detrimental and in fact, a very minor proportion of variants were homologous to deleterious mutations in other species. Previous studies on allozyme variation in the turbot supported much lower variation for this fraction of protein coding genes than in other flatfishes (~ fivefold lower), unlike the very similar diversity observed with microsatellites, which was interpreted as an ancient bottleneck in this species^11^. If this observation could be extrapolated to all protein coding genes, this would mean that a much higher NSV would occur in other flatfish, which is supported by the ~ 10 million SNPs detected in Senegalese sole vs ~ 3 million SNPs in turbot obtained from the recent whole genome resequencing of 12 sole individuals^86^.

The broad NSV collection identified in the turbot was filtered using technical, population genetics and functional information to obtain a consistent database that could be further validated and eventually used with practical purposes on breeding programs and management of wild fisheries. We were very conservative to retain NSVs on functional genes, and those genes with ≥ 3 NSVs were dismissed, which dramatically dropped NSVs to ~ 10,000. We are aware that this filtering is likely very strict and a significant quantity of genes with ≥ 3 NSVs could be functional, so the whole ≥ 50,000 should be considered as a suggestive repository for future studies. Pseudogene identification in the turbot genome using the vast functional information coming from the AQUAFAANG project^87^ will improve our ability to discriminate pseudogenes, resulting in a more refined list of NSVs. The second most important drop (from ~ 10,000 to ~ 1200) was related to previous functional information (differentially expressed genes in response to pathogen challenges or growth) or association (close to QTL for growth and resistance to pathologies) studies in farm populations^28,31^ or with signals of selection related to environmental variables (temperature, salinity) in the wild across its distribution range^30^. The broad genomic information in turbot facilitated the targeting of this subset of NSVs on candidate genes under selection. However, the greater relevance of resistance to pathologies and growth for industry determined a bias in the final selection. Our list includes other interesting genes potentially related to adaptation in the wild (i.e. eight opsin genes very relevant for adaptation to the sea bottom^88^) to be explored in future studies. From this collection, a small subset of NSVs was validated using the MassARRAY genotyping technology on representative wild and farm samples trying to obtain some clues on their relevance for adaptation across its distribution range or in breeding programs. All the 18 SNPs finally genotyped in a single multiplex matched with the in silico predictions supporting the confidence of our pipeline and showed a very robust genotyping with hardly missing data, which makes feasible its further application as a cost-effective molecular tool.

We intended to identify signals of selection in this NSV set, either divergent or stabilizing, in the different scenarios studied using wild and farm populations covering the whole population range of the species and broodstock from companies with breeding programs, respectively. The joint analysis of loci under selection would blur/mask potential population structuration considering the different evolutionary forces^89^, which include clinal, patchiness or local variation patterns involving balanced or divergent selection models. However, locus-specific patterns of spatial variation were observed in the wild, as expected given the environmental variation (both biotic and abiotic) across the turbot distribution range. The most consistent pattens of turbot spatial structure were related to differentiation of the Southern populations at several loci (*fga-like*, *slc12a3*) or specifically in the Black Sea (*eya3*, *hamp*) or the Adriatic Sea (*tshr*), but gradual changes in the Atlantic from the Baltic Sea east and southwards (*ciart*, *LOC118312496*) and very particular local patterns such as *virp1*, were also observed. At the other end, *paxbp1* showed a great constancy across the whole distribution area. Interestingly, some of these gene markers have been associated with strong genetic differentiation at spatial scale in other fish species, like *slc12a3* and *tshr* related to osmoregulation and variation in spawning time, respectively, between Atlantic and Baltic herring^90,91^. Signals of selection for some of these genes has also been reported in other fish species across geographical ranges, such as *paxbp1* linked to myogenesis and thermogenesis^92^, or *virp1* associated with local adaptations to extreme environments^93^.

In addition to *eya3* and *tshr*, outlined before, three other loci, *aqp8b*, *igfbp2* and *hbαD*, showed consistent or suggestive signals of selection when comparing wild vs farm populations. Of note, *igfbp2* and *hbαD* showed a very strong differentiation when comparing wild vs farm populations (F_ST_ > 0.3) due to the increase of a rare allelic variant in the wild in both farms. This fact was not observed in farm 2, which could suggest different selective pressures, or alternatively, a founder effect in the farm 2 broodstock. Interestingly, farms 1 and 3 appeared to be genetically closer (average F_ST_ = 0.015) with regard to farm 2 (F_ST (1 vs 2)_ = 0.030; F_ST (2 vs 3)_ = 0.036), either by historical connection or because similar management protocols or targets of selection are followed.

We looked for additional support to the signals of selection detected by analyzing the consequences of the NSVs detected on the 3D protein structure that could refine their function according to environmental variation. For this, the complementary approaches using protein models of related species and de novo models supported by artificial intelligence tools provided information on the putative action of selection on growth, circadian rhythm and osmoregulation related genes. Furthermore, we also explored functional changes of other NSVs regarding previous information in turbot or in other species to ascertain their putative role on adaptation not evidenced in our population genomics analyses.

IGF-I and IGF-II are important regulators of vertebrate growth and development, and their respective coding turbot genes display distinct expression patterns during metamorphosis^94^. The present turbot study revealed polymorphisms in both the IGF binding protein IGFBP2 and the receptor IGF1R. The binding proteins have a higher affinity for IGF than the receptors and can inhibit and/or enhance IGF actions depending on the physiological context^95^. Teleost fish possess multiple *igfbp* genes of which *igfbp2* encodes a growth inhibitory protein^96^. A polymorphism in the chicken *igfbp2* has been found to be associated with growth and body composition^97^. The Pro264Ser polymorphism of turbot IGFBP2 is positioned in the C-terminal domain, which in human IGFBP2 contributes to IGF-1 binding^98^. The C-terminus including Pro264 is highly conserved in teleost IGFBP2 and was monomorphic in all wild turbot populations examined, except for the Spanish west coast population and farm1 and farm3 that displayed the rare Ser264 variant. Most of the current turbot broodstock have originally been recruited from Spanish and French coasts^18,99^, which could explain the presence of the rare IGFBP2b variant in farmed turbot, but its presence could also be connected to selection for growth considering that this is the main target of breeding programs. Similarly, *igf1rb* showed the highest polymorphism in farm1 and farm3, while the alternative allele was missing in the Baltic Sea, Black Sea and Adriatic Sea. An i*gf1rb* polymorphism was reported to be associated with growth traits in the freshwater goby *Odontobutis potamophila*^100^, and divergence and polymorphism analysis of *igf1ra* and *igf1rb* in the orange-spotted grouper (*Epinephelus coioides*) suggested their importance in growth regulation and breeding of this species^101^. Moreover, the involvement of *igf1rb* in growth during hypoxia was recently reported in a genome-wide association analysis of adaptation to oxygen stress in farmed Nile tilapia (*Oreochromis niloticus*)^102^. Our study revealed a very strong differentiation of the polymorphic HbαD subunit when comparing wild vs farm populations. We predict that the Thr44 variant identified in farm1 and farm3 increases the oxygen binding affinity similar to the human hemoglobin Kawachi (Pro44α → Arg) variant^103^ of importance during hypoxic conditions.

PAXBP1 is involved in skeletal muscle formation by linking the transcription factors PAX3 and PAX7 on chromatin to regulate the muscle progenitor cells proliferation. The pathogenic human variant Arg538Cys underlies syndrome of global developmental delay and myopathic hypotonia^104^, while the significant of the Pro47Leu substitution in turbot PAXBP1 is unknown.

Turbot is an active visual predator and shows circadian cycles of locomotor and food anticipatory activities together with rhythmic expression of core circadian clock genes^105^. Among the polymorphic turbot genes displaying high allelic diversity, we identified *tshr, eya3* and *ciart*, which are involved in the regulation of circadian and seasonal rhythms. TSHR plays an important role in seasonal reproduction through the conserved EYA3-TSH pathway^106,107^. Polymorphisms in herring TSHR were shown to contribute to the regulation of spring or autumn spawning^36^, while the Leu339Glu polymorphism in turbot TSHR is positioned in a flexible region. Such intrinsically disordered regions are common in eukaryotic proteins and important biological functions have been associated with them, such as flexible linker, cellular signal transduction, protein phosphorylation^108,109^. It has been observed that function can arise directly from the disordered state whereas in other cases their function originates from binding-induced folding promoted by other proteins or RNA, DNA molecules^110^. Evidence of EYA3 as an integrator of photoperiodic cues and nutritional regulation was recently found in Atlantic cod (*Gadus morhua*)^111^. The Ser230Gly substitution in turbot EYA3 is positioned in the PST (Pro-Ser-Thr)-rich domain necessary for transcriptional activity of *Drosophila* EYA^112,113^, while both Ser and Gly were identified at the corresponding site in various teleost. The circadian-associated transcriptional repressor CIART is involved in the eye regression of cave molly (*Poecilia mexicana*)^114^, whereas turbot *ciart* proved to be differentially expressed in freshwater- *versus* seawater-acclimated fish^115^. A missense polymorphism in pig *ciart* was reported to be associated with backfat thickness^116^. Both the Asn and Ser residues in the polymorphic position 271 of turbot CIART are found in other teleost.

The important role played by the kidney in the osmoregulatory response of turbot to low salinity has been examined by transcriptome analysis^25,115^. SLC12A3, or the Na + Cl–cotransporter NCC1 paralog, is highly expressed in the kidney of fish acclimated to freshwater and is crucial for the ion reabsorption in the collecting duct^117^. Turbot *slc12a3* showed highest polymorphic diversity in the Black Sea and Adriatic Sea, in contrast to the Baltic Sea. We noted that the Cys residue at position 938 in turbot *slc2a3* is novel among marine fish, except for Antarctic fish. *aqp8b* is highly expressed in fish kidney tubuli serving as important pathways for reabsorbed water^118^. Turbot *aqp8* was only polymorphic in the Spanish west coast population and in farm1 and farm3 as outlined before for *igfbp2*. The acidic Gln residue at position 36 is invariable in teleost AQP8, and the basic His replacement together with the novel Cys938 variant of Slc2a3 await further studies.

Turbot *vipr1b* showed high polymorphic diversity in both wild populations and farms examined, except in the Baltic Sea and Spanish west coast. A conserved role of the VIP neuropeptide in the immune system and inflammatory processes in olive flounder (*Paralichthys olivaceus*) was suggested by the significant changes in *vip* mRNA levels in spleen and head kidney when exposed to an artificial bacterial challenge by *Edwardsiella tarda*^119^. VIP binds to the N-terminal end of the receptor, which in turbot contains an Asn2Gln polymorphism. VIPR1 polymorphism has been linked to gastrointestinal dysmotility disorders in man^120^, but associated with reproductive traits in birds^121^*.* Two polymorphic hepcidins have been identified in turbot^122^ of which *hep1* was highly polymorphic in all populations and farms examined, particularly in the Black Sea and farm 3. The Asn81Tyr substitution is positioned in the mature peptide, but it does not seem to affect the conserved Cys residues as shown by the polymorphic *hep2*^122^. Both *hep1* and *hep2* possess antimicrobial activity and promote resistance against bacterial and viral infection, but the antimicrobial activities of *hep2* were significantly stronger than those of *hep1 *in vitro and *in vivo*^123^. However, only *hep1* was upregulated after iron overloading that is consistent with the presence of a hypothetical iron regulatory sequence, which is lacking in *hep2*^123^.

## Conclusions

We constructed the first atlas of NSVs in the turbot genome and designed a conservative pipeline to define a robust dataset that could be further validated for their implication on adaptation in the wild or farm conditions using population genomics or 3D functional approaches. This strategy enabled the identification of consistent or suggestive signals of selection related to growth, osmoregulation, hypoxia or immunity that might be further applied for functional and association studies using a robust and cost-effective genotyping methodology. Our study does not only provides a suitable strategy for turbot, but it could be expanded to other fish species considering the increasing genomic resources available in public databases.

## Supplementary Information

Supplementary Figure S1.

Supplementary Table S1.

Supplementary Table S2.

Supplementary Table S3.

Supplementary Table S4.

Supplementary Table S5.

Supplementary Table S6.

## Data Availability

Resequencing data of five males and five females are available at NCBI databases BioProject PRJNA649485 (https://www.ncbi.nlm.nih.gov/bioproject/649485), accession number SRX8843737. Genotyping data used in this study is provided in Table S5 and the primer sets for SNP genotyping included in Table S4.
