# Supplementary material for: Non-synonymous variation and protein structure of candidate genes associated with selection in farm and wild populations of turbot (Scophthalmus maximus)
Source: Sci Rep. 2023 Feb 21;13:3019. doi: 10.1038/s41598-023-29826-z (PMC9944912; doi:10.1038/s41598-023-29826-z)
Supplement: Supplementary file 1 — Supplementary Figure S1. [file 41598_2023_29826_MOESM1_ESM.pdf]

Supplementary figure S1. Allele frequency variation in the 18 candidate genes examined across the whole distribution range and in the main farm broodstock of turbot (*Scophthalmus maximus*).

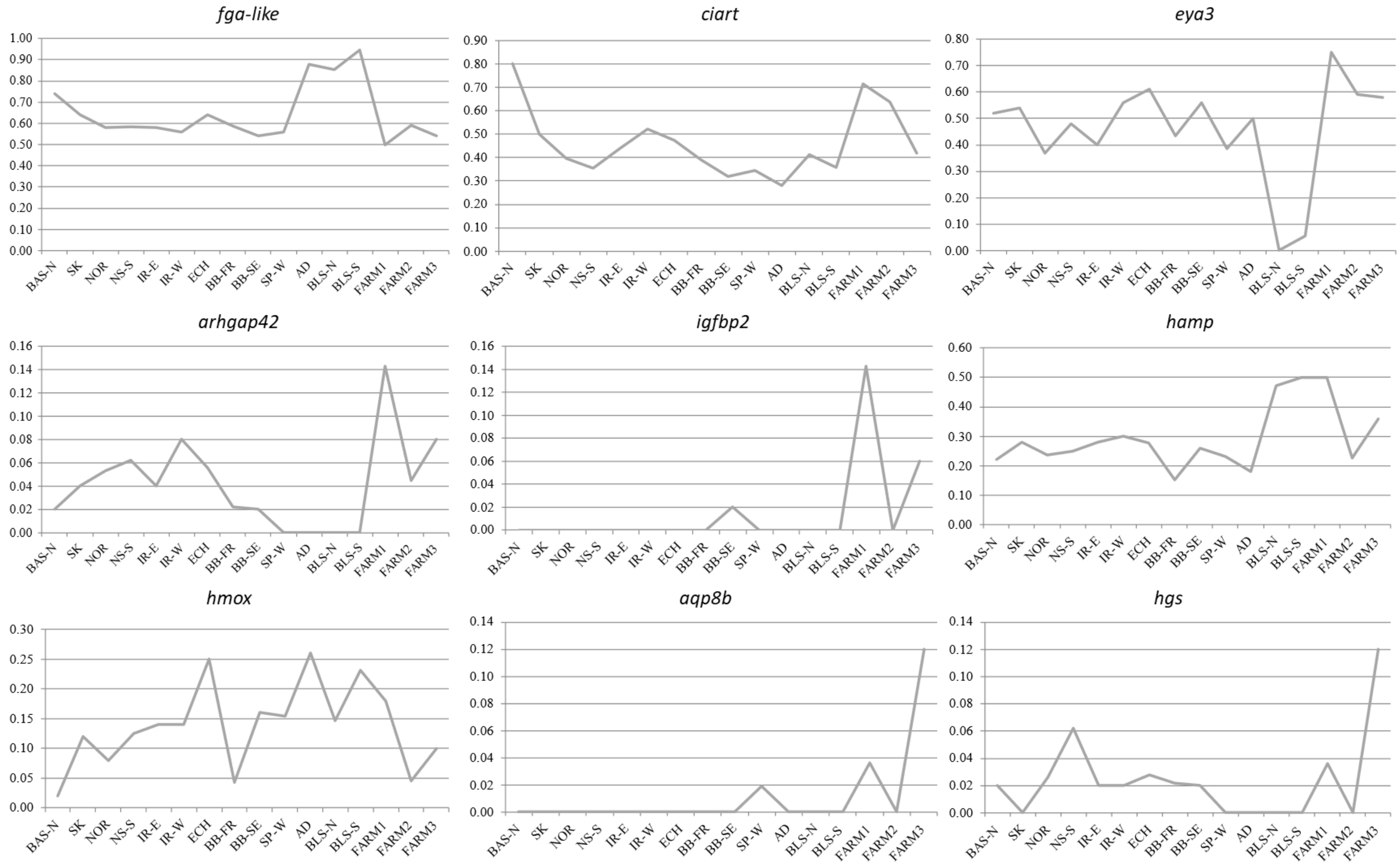

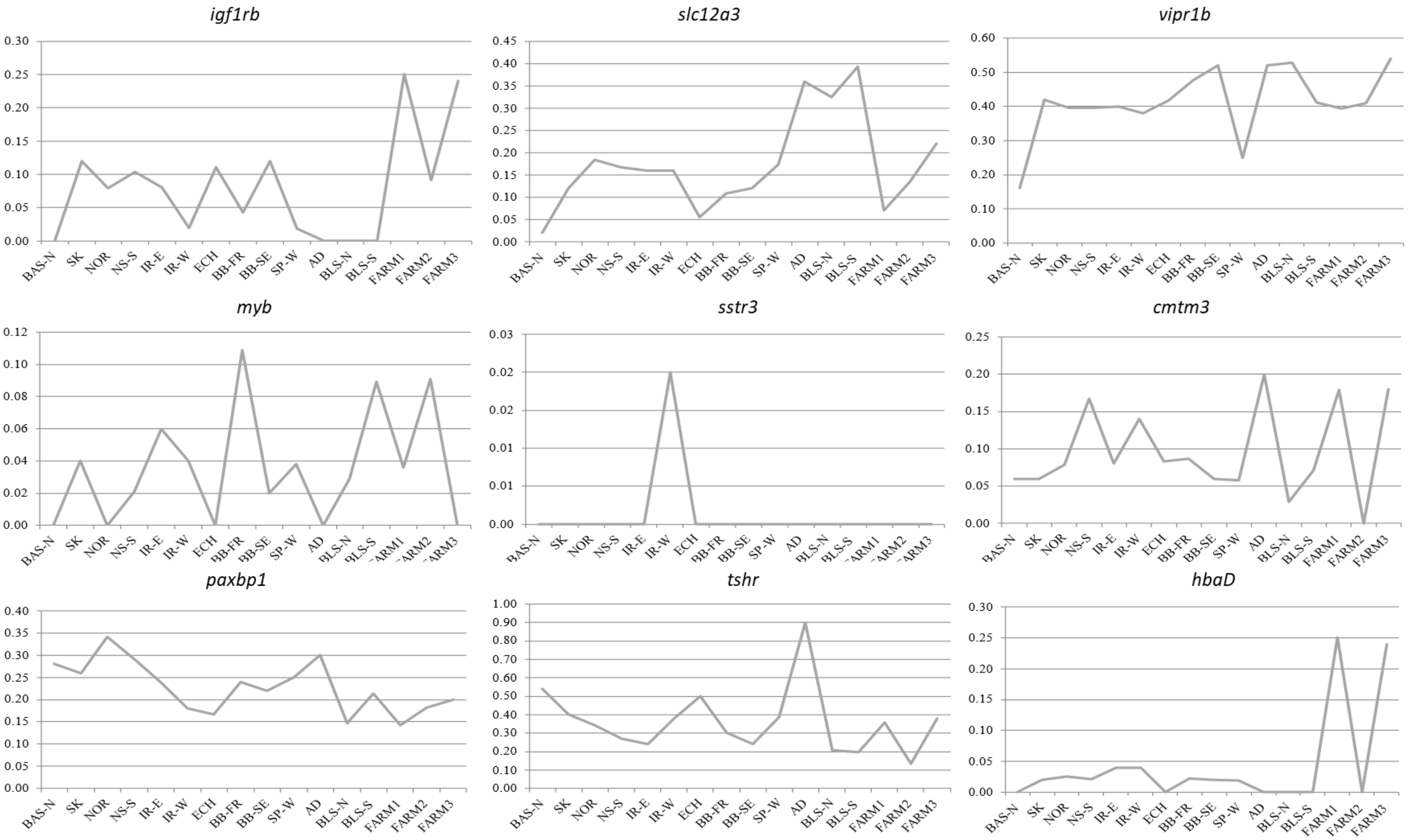
igf1rb
0.30
0.25
0.20
0.15
0.10
0.05
0.00
BAS-N SK NOR NS-S IR-E IR-W ECH BB-FR BB-SE SP-W AD BLS-N BLS-S FARM1 FARM2 FARM3
slc12a3
0.45
0.40
0.35
0.30
0.25
0.20
0.15
0.10
0.05
0.00
BAS-N SK NOR NS-S IR-E IR-W ECH BB-FR BB-SE SP-W AD BLS-N BLS-S FARM1 FARM2 FARM3
vipr1b
0.60
0.50
0.40
0.30
0.20
0.10
0.00
BAS-N SK NOR NS-S IR-E IR-W ECH BB-FR BB-SE SP-W AD BLS-N BLS-S FARM1 FARM2 FARM3
myb
0.12
0.10
0.08
0.06
0.04
0.02
0.00
BAS-N SK NOR NS-S IR-E IR-W ECH BB-FR BB-SE SP-W AD BLS-N BLS-S FARM1 FARM2 FARM3
sstr3
0.03
0.02
0.02
0.01
0.01
0.00
BAS-N SK NOR NS-S IR-E IR-W ECH BB-FR BB-SE SP-W AD BLS-N BLS-S FARM1 FARM2 FARM3
cmtm3
0.25
0.20
0.15
0.10
0.05
0.00
BAS-N SK NOR NS-S IR-E IR-W ECH BB-FR BB-SE SP-W AD BLS-N BLS-S FARM1 FARM2 FARM3
paxbp1
0.40
0.35
0.30
0.25
0.20
0.15
0.10
0.05
0.00
BAS-N SK NOR NS-S IR-E IR-W ECH BB-FR BB-SE SP-W AD BLS-N BLS-S FARM1 FARM2 FARM3
tshr
1.00
0.90
0.80
0.70
0.60
0.50
0.40
0.30
0.20
0.10
0.00
BAS-N SK NOR NS-S IR-E IR-W ECH BB-FR BB-SE SP-W AD BLS-N BLS-S FARM1 FARM2 FARM3
hbaD
0.30
0.25
0.20
0.15
0.10
0.05
0.00
BAS-N SK NOR NS-S IR-E IR-W ECH BB-FR BB-SE SP-W AD BLS-N BLS-S FARM1 FARM2 FARM3
