# Supplementary material for: Non-synonymous variation and protein structure of candidate genes associated with selection in farm and wild populations of turbot (Scophthalmus maximus)
Source: Sci Rep. 2023 Feb 21;13:3019. doi: 10.1038/s41598-023-29826-z (PMC9944912; doi:10.1038/s41598-023-29826-z)
Supplement: Supplementary file 3 — Supplementary Table S2. [file 41598_2023_29826_MOESM3_ESM.pdf]

Table S2. List of NSVs of *Scophthalmus maximus* detected in other species using PROVEAN software (Choi and Chan, 2015) and their categorization according to selection criteria (deleterious vs neutral)

Gene name	Protein ID	aa change	PROVEAN score	Prediction (cutoff = -2.5)	Number of sequences
acaa2	>XP_035494648.1 3-ketoacyl-CoA thiolase, mitochondrial [Scophthalmus maximus]	Q188H	-3717	Deleterious	122
aldoab	>XP_035467885.1 aldolase a, fructose-bisphosphate, b [Scophthalmus maximus]	G102A	-5026	Deleterious	602
cry-dash	>XP_035474576.1 cryptochrome DASH [Scophthalmus maximus]	E109del	-9.343	Deleterious	55
pomc-like	>XP_035474322.1 pro-opiomelanocortin-like [Scophthalmus maximus]	P137R	-7 476	Deleterious	1225
aqp8b	>XP_035471338.1 aquaporin-8b [Scophthalmus maximus]	Q36H	-3 422	Deleterious	108
clic2	>XP_035505125.1 chloride intracellular channel protein 2 isoform X3 [Scophthalmus maximus]	S126G	-2 923	Deleterious	202
samhd1-like	>XP_035501047.1 LOW QUALITY PROTEIN: deoxynucleoside triphosphate triphosphohydrolase SAMHD1-like [Scophthalmus ma	Q262L	-3,691	Deleterious	113
paxbp1	>XP_035480876.1 PAX3- and PAX7-binding protein 1 [Scophthalmus maximus]	Y406S	-3,612	Deleterious	141
angptl3	>XP_035504482.1 angiopoietin-related protein 3 isoform X1 [Scophthalmus maximus]	D216E	0.428	Neutral	128
angptl4	>XP_035490470.1 angiopoietin-related protein 4 [Scophthalmus maximus]	C11S	-1504	Neutral	237
angptl7	>XP_035500760.1 angiopoietin-related protein 7 [Scophthalmus maximus]	S7G	-0.687	Neutral	155
ciart	>XP_035497547.1 circadian-associated transcriptional repressor [Scophthalmus maximus]	N271S	1.000	Neutral	45
cops4	>XP_035481315.1 COP9 signalosome complex subunit 4 isoform X3 [Scophthalmus maximus]	G382del	1.743	Neutral	133
crhbp	>XP_035483008.1 corticotropin-releasing factor-binding protein [Scophthalmus maximus]	A28P	-0.471	Neutral	91
dner	>XP_035479717.1 delta and Notch-like epidermal growth factor-related receptor [Scophthalmus maximus]	T55N	-0,615	Neutral	143
enox2	>XP_035505029.1 ecto-NOX disulfide-thiol exchanger 2 isoform X1 [Scophthalmus maximus]	A570T	0.080	Neutral	174
enox2	>XP_035505031.1 ecto-NOX disulfide-thiol exchanger 2 isoform X1 [Scophthalmus maximus]	A570T	-0.033	Neutral	146
eya3	>XP_035476747.1 eyes absent homolog 3 [Scophthalmus maximus]	R22L	0.468	Neutral	330
eya3	>XP_035476747.1 eyes absent homolog 3 [Scophthalmus maximus]	S230G	-0.461	Neutral	330
fgf12a	>XP_035490418.1 fibroblast growth factor 12a isoform X4 [Scophthalmus maximus]	R161C	-1.685	Neutral	307
slc2a9	>XP_035486108.1 solute carrier family 2, facilitated glucose transporter member 9-like isoform X1 [Scophthalmus maximus]	I317L	-0.078	Neutral	166
slc2a9	>XP_035486109.1 solute carrier family 2, facilitated glucose transporter member 9-like isoform X2 [Scophthalmus maximus]	I317L	0.254	Neutral	136
sstr3	>XP_035471741.1 somatostatin receptor type 5 [Scophthalmus maximus]	S414L	0.135	Neutral	229
sstr3	>XP_035471742.1 somatostatin receptor type 5 [Scophthalmus maximus]	S414L	0.135	Neutral	229
tshr	>XP_035472836.1 thyrotropin receptor [Scophthalmus maximus]	L339Q	0.202	Neutral	434
myb	>XP_035474017.1 transcriptional activator Myb isoform X1 [Scophthalmus maximus]	C4Y	-0.218	Neutral	249
myb	>XP_035474018.1 transcriptional activator Myb isoform X2 [Scophthalmus maximus]	C4Y	-0.483	Neutral	221
tgfb2	>XP_035464164.1 transforming growth factor beta-2 proprotein isoform X1 [Scophthalmus maximus]	K49E	-1 995	Neutral	206
tgfb2	>XP_035464165.1 transforming growth factor beta-2 proprotein isoform X1 [Scophthalmus maximus]	K49E	-1 995	Neutral	206
tgfb2	>XP_035464166.1 transforming growth factor beta-2 proprotein isoform X2 [Scophthalmus maximus]	K49E	-2 089	Neutral	153
tgfb2	>XP_035464167.1 transforming growth factor beta-2 proprotein isoform X3 [Scophthalmus maximus]	K49E	-2 156	Neutral	200
tgfb2	>XP_035464168.1 transforming growth factor beta-2 proprotein isoform X4 [Scophthalmus maximus]	K49E	-1 971	Neutral	215
vipr1b	>XP_035475663.1 vasoactive intestinal polypeptide receptor 1b [Scophthalmus maximus]	N2D	-0.061	Neutral	244
hacd3	>XP_035498675.1 very-long-chain (3R)-3-hydroxyacyl-CoA dehydratase [Scophthalmus maximus]	V226I	0.224	Neutral	100
hacd3	>XP_035498675.1 very-long-chain (3R)-3-hydroxyacyl-CoA dehydratase [Scophthalmus maximus]	H44Y	-2 236	Neutral	100
vtna	>XP_035478316.1 vitronectin a [Scophthalmus maximus]	D185E	0.579	Neutral	78
slc4a3	>XP_035506973.1 anion exchange protein 3 isoform X1 [Scophthalmus maximus]	L212F	0.152	Neutral	235
slc4a3	>XP_035506973.1 anion exchange protein 3 isoform X1 [Scophthalmus maximus]	S39N	0.380	Neutral	235
slc4a3	>XP_035506974.1 anion exchange protein 3 isoform X2 [Scophthalmus maximus]	L212F	-1 020	Neutral	236
cldn14	>XP_035459517.1 claudin-14-like [Scophthalmus maximus]	R198G	-1,146	Neutral	202
cldn18	>XP_035490297.1 claudin-18 [Scophthalmus maximus]	H209Q	0,163	Neutral	235
cftr	>XP_035497382.1 cystic fibrosis transmembrane conductance regulator isoform X1 [Scophthalmus maximus]	Q1263E	0,008	Neutral	216
ncx-like	>XP_035482861.1 sodium/calcium exchanger 2-like isoform X1 [Scophthalmus maximus]	D683N	0,340	Neutral	798
ncx-like	>XP_035482861.1 sodium/calcium exchanger 2-like isoform X1 [Scophthalmus maximus]	V331M	0,287	Neutral	798
slc28a1	>XP_035497155.1 sodium/nucleoside cotransporter 1 isoform X1 [Scophthalmus maximus]	I145M	-0,035	Neutral	148
slc28a1	>XP_035497155.1 sodium/nucleoside cotransporter 1 isoform X1 [Scophthalmus maximus]	M636L	0,284	Neutral	148
slc12a3	>XP_035497742.1 solute carrier family 12 member 3 [Scophthalmus maximus]	D38N	-0,172	Neutral	256
slc12a3	>XP_035497742.1 solute carrier family 12 member 3 [Scophthalmus maximus]	C938S	1,354	Neutral	256
acss3	>acss3-201 peptide: ENSSMAP00000007258 pep:protein_coding	G56V	-1,475	Neutral	143
acss3	>acss3-201 peptide: ENSSMAP00000007258 pep:protein_coding	V23A	-0,061	Neutral	143
ccnb1	>ccnb1-201 peptide: ENSSMAP00000023155 pep:protein_coding	A390V	-1,871	Neutral	176
cmtm3	>cmtm3-201 peptide: ENSSMAP00000034923 pep:protein_coding	K83R	-0,845	Neutral	109
dpm1	>dpm1-201 peptide: ENSSMAP00000003754 pep:protein_coding	G18E	1,442	Neutral	250
erf	>XP_035476304.1 LOW QUALITY PROTEIN: ETS domain-containing transcription factor ERF [Scophthalmus maximus]	H328P	-1,217	Neutral	131
fgf1a	>fgf1a-201 peptide: ENSSMAP00000008030 pep:protein_coding	P91L	-1,592	Neutral	332
g6pc3	>g6pc3-201 peptide: ENSSMAP00000013142 pep:protein_coding	L10F	0,173	Neutral	162
igf1rb	>XP_035483940.1 insulin-like growth factor 1b receptor isoform X2 [Scophthalmus maximus]	Y980H	-1,01	Neutral	210
fga-like	>XP_035462489.1 fibrinogen alpha chain-like isoform X2 [Scophthalmus maximus]	R537Q	1,849	Neutral	165
fga-like	>XP_035462489.1 fibrinogen alpha chain-like isoform X2 [Scophthalmus maximus]	A574S	-1,321	Neutral	165
fga-like	>XP_035467788.1 heterogeneous nuclear ribonucleoprotein C-like isoform X1 [Scophthalmus maximus]	A139T	-0,187	Neutral	275
arhgap42	>XP_035479846.1 rho GTPase-activating protein 42 isoform X1 [Scophthalmus maximus]	T632K	-0,614	Neutral	238
arhgap42	>XP_035479847.1 rho GTPase-activating protein 42 isoform X2 [Scophthalmus maximus]	T793N	-0,498	Neutral	239
frs2	>XP_035499280.1 LOW QUALITY PROTEIN: fibroblast growth factor receptor substrate 2-like [Scophthalmus maximus]	A124T	0,431	Neutral	95
frs2	>XP_035499280.1 LOW QUALITY PROTEIN: fibroblast growth factor receptor substrate 2-like [Scophthalmus maximus]	A290P	-0,25	Neutral	95
samhd1-like	>XP_035501047.1 LOW QUALITY PROTEIN: deoxynucleoside triphosphate triphosphohydrolase SAMHD1-like [Scophthalmus ma	P253H	-0,871	Neutral	113
mtmr7b-201	>mtmr7b-201 peptide: ENSSMAP00000015282 pep:protein_coding	R454G	-1,136	Neutral	152
phka2	>XP_035483685.1 LOW QUALITY PROTEIN: phosphorylase b kinase regulatory subunit alpha, liver isoform [Scophthalmus maxin	P1050L	-0,489	Neutral	172
rnf4	>XP_035505177.1 E3 ubiquitin-protein ligase RNF4 isoform X1 [Scophthalmus maximus]	D55N	-0,975	Neutral	97
rnf4	>XP_035505183.1 E3 ubiquitin-protein ligase RNF4 isoform X3 [Scophthalmus maximus]	G6S	1,253	Neutral	97
Rsl1d1	>XP_035468633.1 ribosomal L1 domain-containing protein 1 [Scophthalmus maximus]	K363N	-2,1	Neutral	79
fgfr3	>XP_035466254.1 fibroblast growth factor receptor 3 isoform X1 [Scophthalmus maximus]	P45R	1,229	Neutral	449
g6pc3	>XP_035468288.1 glucose-6-phosphatase 3 [Scophthalmus maximus]	L10F	0,173	Neutral	162
hspb7	>XP_035501243.1 heat shock protein beta-7 [Scophthalmus maximus]	A56S	-0,005	Neutral	144
hspb7	>XP_035501243.1 heat shock protein beta-7 [Scophthalmus maximus]	S41T	-0,945	Neutral	144
hbaD	>XP_035493027.1 heme oxygenase-like [Scophthalmus maximus]	T81A	0,075	Neutral	153
hbaD	>XP_035469583.1 hemoglobin subunit alpha-D [Scophthalmus maximus]	A44T	-0,248	Neutral	752
hdaD	>XP_035469583.1 hemoglobin subunit alpha-D [Scophthalmus maximus]	V78I	-0,731	Neutral	752
hpxa	>XP_035507395.1 hemopexin [Scophthalmus maximus]	A8V	1,081	Neutral	96
hpxa	>XP_035507395.1 hemopexin [Scophthalmus maximus]	A383T	-1,977	Neutral	96
hgs	>XP_035471064.1 hepatocyte growth factor-regulated tyrosine kinase substrate isoform X2 [Scophthalmus maximus]	P726T	-0,52	Neutral	113
hamp	>XP_035496795.1 hepcidin-1 [Scophthalmus maximus]	N81Y	-1,483	Neutral	137
igfbp2	>ADW77214.1 insulin-like growth factor binding protein 2 [Scophthalmus maximus]	P264S	-0,464	Neutral	206
paxbp1	>XP_035480876.1 PAX3- and PAX7-binding protein 1 [Scophthalmus maximus]	P47L	-0,861	Neutral	141
ppargc1a	>XP_035504838.1 peroxisome proliferator-activated receptor gamma coactivator 1-alpha isoform X1 [Scophthalmus maximus]	L540V	0,255	Neutral	172
