# Supplementary material for: Non-synonymous variation and protein structure of candidate genes associated with selection in farm and wild populations of turbot (Scophthalmus maximus)
Source: Sci Rep. 2023 Feb 21;13:3019. doi: 10.1038/s41598-023-29826-z (PMC9944912; doi:10.1038/s41598-023-29826-z)
Supplement: Supplementary file 4 — Supplementary Table S3. [file 41598_2023_29826_MOESM4_ESM.pdf]

Table S3: Genomic position and adjacent sequences of the SNPs selected for the Sequenom multiplex on candidate genes associated with growth, osmoregulation and resistance to diseases in turbot (*Scophthalmus maximus*)

>4 dna:primary_assembly
primary_assembly:ASM1334776v1:4:10761572:10761772:1[SNP_9677][**paxbp1**]
AGTTTCCTTCTTCGCGGCTCTACCACCGCTGAGGTTTGCCAGAAAGCCGTTGCTGTTGAAGTTGTCCGAGGCGCCGGGTGCGCGGGTAA
AGGCGCTGGTG[G/A]GATTCTCCATGAACGGGATTTCCTCCACCACGGGCCCGCACGACGTCGGCGCCAGCGGGGGCCGCTGACTCGCC
TCCGGCTCGTCCTCGTCGGATTCATT
>5 dna:primary_assembly
primary_assembly:ASM1334776v1:5:22619662:22619862:1[SNP_13507][**igf1rb**]
TAAATCGAGCCTCCGTGTGTCCGTCTCACCAGCGTTCATCAGTTCGAGGACATGGTGAGTCGCTTCAGGCTGGACACCAACCCCAAGCA
GGACTCTGATT[G/C]TGGGCCGCCGGGCCAGCGGGAGGGGCCCTGGATGATCTCTGAGGAGGATGTGGAGAGGAACAAGGCGAAGGTC
GGAATACCACGCACGCAAACTCACCGA
>10 dna:primary_assembly
primary_assembly:ASM1334776v1:10:24208741:24208941:1[SNP_28230][**slc12a3**]
AGTGTCCAGCGACGTCATTCACCTGGCTGCATTCAGACCCAGAGCTCCTCCGCCAGTCGTCAATGGGAAGGCAACACAAGGCGGGGGG
TTGAACGAGTCC[G/A]ATTACTATCAGTGTCTCCGCGACGGGGGCATCGTTGCGCCCCCTGGATCCGATGCTCCGACCGGCTACGAGACT
CTGGACTCTCCGCCGCACTACGACTT
>14 dna:primary_assembly
primary_assembly:ASM1334776v1:14:4200879:4201079:1[SNP_35978][**LOC118320384**]
TTACATGTTGATAGAACCACCCAATCAATAGAAGTTGGAAGAAAACCCAAATCTGTCCCGATGTTTGCTGAGCTGTGCTAGAGTGATGT
GGCTGCAGTGG[G/A]CATTTCTCTGAACTCCTCTTGAAACTGGTTACAGTTGGGGTCCCCTCTGACTTTAGGTGACGCTGGTATCTGGACC
CCAGTTAAGGGGTTAACGCACCAG
>18 dna:primary_assembly
primary_assembly:ASM1334776v1:18:9552404:9552604:1[SNP_45672][**zgc:163057**]
CCACCTGGACATCAGTGCCCGCTCCGCTCACCTGCTCTCCCACGGGAAGAAGATTGTCCTGGCCATAGCAGAGGGAGCCAAAGACATCA
GCCAGCTGACC[G/A]TCACCCTGGCTCCTCTGCAAACAATGCACGCCTACCAGCTCCGGATAGACCCGACGAACTTCAAGGTGCAGGTC
CAGGAAACAGAGAAGCAATTCTACAC
>19 dna:primary_assembly
primary_assembly:ASM1334776v1:19:19867048:19867248:1[SNP_49229][**sstr3**]
GACGAGGAGGACGAAGACGTCTCCGAGATGACGGAGATCTACAGAATCGCCCAGAACGGAAACAGCAGTTTCCAGCCGCAGAGCTCCC
GGCAGCTCTTGT[C/T]GGAGAGAGGACCGACTCCAGGAGCGGCGGAGCTGCCGTCCCCGGACAACAAGGACAAAGCCGGGGATGCGAG
CGGGAAAGATCCCGCCAACGGGTCCACG
>22 dna:primary_assembly
primary_assembly:ASM1334776v1:22:5580911:5581111:1[SNP_53785][**eya3**]

GATGTCCGTCACAGTCCTGAAGCAGCAGAGATAATCAAATTATGTCGAAAAAAAATCTATAAACACGTTTTTGTGTGTATGCAGGCAAA
GAAAGCCAAGC[G/T]GGAGATAGATGGTGGACTGGAGAAAGAGTCATGGTTGGTCATTTCTGATCTTTTCATTCTTTTAACTCGCTTATA
GTGTCTGATCGCTGGCAGTTGCCGC
>2 dna:primary_assembly
primary_assembly:ASM1334776v1:2:1117514:1117714:1[SNP_3450][**LOC118284026**]
CCTGTTGACGAGTCGGGGCGAGACCATGCTGAAAGTGGAGCTTGAGGATTGGGAGGGGGGCGTGGCCGGCGCCGAGTACATGATAAGA
GTTGGTTCGGAG[G/T]CGGAGGGGTTCCCGCTGCATGTGTCAGGGTACACGGGGGACGCCGGCGACGCGCTGACGATGCCCAAGTCCGA
CACGGCGTCTTACCTGATCCACAACGG
>3 dna:primary_assembly
primary_assembly:ASM1334776v1:3:7594519:7594719:1[SNP_6462][**LOC118299874**]
AGGACGCCCTGACTCTGACTCAGCTCTGCCTTGTATAGGGGGTTGGCATACTCGCCTCTGTCCTTCCACAAATGAGGCCATAGAGAGTG
ACTCCGCTCAC[G/C]AAGCCTGCATCCACACACGCACGCACACACACACAAACACACAGTCAAGTCAAGTCAAGGAACTCAAAGGACTC
CGCTCCCGTCATAGCAGCTGGCAGAC
>10 dna:primary_assembly
primary_assembly:ASM1334776v1:10:12737908:12738108:1[SNP_26879][**LOC118314898**
]
GCCGCTGCAGCCGCGAGCCCCGTCCCCGTGAGCGATCCTACAGTCGGCGCCAAAGCCCTGAGAGTCACCGACCTCACTCCCGGTGGAGC
CTACTCCTCCA[A/G]TAACCTGAAGTGGCTCCACACATCACCCATCTGCTCCCCCACAGCAGAGCAGGCCCAGGCTGGCCCCAGGCACCT
GCTGGCCCCCAGAGACAGAGACCTA
>10 dna:primary_assembly
primary_assembly:ASM1334776v1:10:25875314:25875514:1[SNP_28575][**LOC118315800**
]
CACCCCTGTCCAGAGGCAGCTGATGGAGAAGGAGAAGAGGCAGCAGGAGCTGACGGAGGATGTGGAGCCACAAGAGACTAACGGCCA
CGTCGAGGCCCCG[G/C]CCGAGCCCGAGCCCGCCGCCCCGCACTCCAACAGCTCCTCCAATCCATCGACGGCTCCTCGTCGCCACCGCCC
GCCCCCCTCACCCCCGACCTGCAGAAT
>1 dna:primary_assembly
primary_assembly:ASM1334776v1:1:14424248:14424448:1[SNP_1344][**hamp**]
TTCACGTCTCTTGTCTCCCACACAGGTGCCGAGTCACATCAGGCAGAAGCGACAGAGCCACATCTCCCTTTGCCGCTGGTGCTGCAACTG
CTGCAAGGCC[A/T]ACAAGGGCTGTGGCTTCTGCTGCAAGTTCTGAGGATAACCCGCAGGAGCCTCGAAATATTAATTTATTGTGCGTTT
TTTTGGGGGGTCATTTCTTGGCCT
>5 dna:primary_assembly
primary_assembly:ASM1334776v1:5:8309596:8309796:1[SNP_12385][***phka2***]
CTGGTAGAAACCCACCGGTACTCTGTTGATGGCTCCATCCAGCCTGCGTCTCCTCAGCCACTGGCCCTGCCTCTCCTCCCACTGCAGCTG
TCCGTCCGCC[G/A]GACCAGGGCCCACTGGGGACAGGATTCCACTGGGGGTGGAGGGGCTGCTGCAGCGCTGTGGTGCCAGAACAGAA
AAAGATGCTGTTAGAAAATGTAGTGG

>5 dna:primary_assembly
primary_assembly:ASM1334776v1:5:10466705:10466905:1[SNP_12604][**cmtm3**]
AAGGAAGAAGGAACACACATTATACAGAGAATTTTTTAAATGATTTAAAAATGTAAAAAAAATAAAATTGTCTCTCACCATGAGAGGC
CAGAGGAAGCCT[T/C]TAAACCTCTCGTTGAATTTGGTGGAGTAAGCAAACAACAAGAAGAGCGCAGCCAGGAACTCCAGCAGCGGGA
CTGTCACGAAGGCCGCGGCCGTGGACGC
>8 dna:primary_assembly
primary_assembly:ASM1334776v1:8:13186874:13187074:1[SNP_21764][**LOC118312496**]
ATTTTATGCCTCTTGAATCACTGCACAGTGTCAACGAGCATGCTTGAAGATGAGTTTTGTTCTATGAAAACTTATTTTCTGATGTTTTAAT
TTCTTCAGG[A/G]CCACAATGGAGACAGAGATGAAGACTCAGACGGCAGCAGAACAGATGACAGACAGGTAGGAATAAGCAGCTGTTA
CCTATTGTATCTCACATCACCAGAG
>16 dna:primary_assembly
primary_assembly:ASM1334776v1:16:2502228:2502428:1[SNP_40700][***ccnb1***]
ATGTAATGCAGTTTTTGTTTCTAACCCCTCCATCTTTACTTCTGTCAGGCCATCAAGAGCAAATACTCCACTTCCAAGCAGATGAGGATC
GCCTCCCTCG[C/T]GCAGCTCAAGTCTTCGGTCGTGAAGGAACCGCGCAGCAGCTCACGCAGTGAGACGGCTGAATATTTCCTTTCTTTT
GGGCACCATGTGCTGATTTGTACA
>17 dna:primary_assembly
primary_assembly:ASM1334776v1:17:2440128:2440328:1[SNP_42914][***fgfr3***]
TCCTGGAATCTCACTGAAGACATTGCCGGTGTGCGTATGACCAGGCAGCACAGACATAAAGCGCCACAGGGCCCGTTGTTGCGTCCTCC
TACCTGTGGGC[G/C]GAGCGGTGGGCAGCCGGGCCGGCGCGACGCACAGAGGCAGGACGACGGAGAGCCAGAGGGAGCACCACAGAG
CCGTCATCCCTCCGGACGGCGCGGCGAG
>19 dna:primary_assembly
primary_assembly:ASM1334776v1:19:13550806:13551006:1[SNP_48518][**hgs**]
TTATATGGGCTACCAGCCCTACGGCATGCAGAACATGATTTCAGCATTGCCAGGACAGGACCCCAATATGCCCCCACAACAGCCGTACA
TGCCAGGCCAG[C/A]CGCCCATGTACCAGCAGGTCAGTTGTTTGTGGGAAATTCGCTTAAAGTCTTTACGTTGATTATTGATCGGTGTCC
ATATCTTTGCTGTTCATTTTCAAGG
>20 dna:primary_assembly
primary_assembly:ASM1334776v1:20:5846927:5847127:1[SNP_49717][**tshr**]
AACCTGACTGTTCTCCAAGACCAGCATCACAAGCGCTCTGTGGGGCCCCTCCGCATGCCTTCCCTCCAAGCGGATAGCGTGGTCGAAAC
AGTCCCAGACC[T/A]GGAGCCAAGTGACGGAGGTCAGAGAGAGTCCCAGCAAGACTGGAGGAGTGGTGACTTCCACGGCAGCCTCCAC
TACCATGCCTACTTTGGGGGCCAGCCA
>20 dna:primary_assembly
primary_assembly:ASM1334776v1:20:10185492:10185692:1[SNP_50073][**myb**]
CATGCTGCTGCTGCTGTGTGTGTGTGTGAAGCGGCTCGCAGGTTTGCGCTGAGCACGTACTTTCGCTCTCGGCTCTGAGTGAGTTCTCCC
GGCGAGGAGG[C/T]AGGTTGACATTAATTTAAACTGCAGCAGGCTCCGCGGCCGCTCCTCCCTCTCTGCTCGCATCCCGGTAAATTTGGC
GCTCCTGATGTGCGCATGCGCAAC

>21 dna:primary_assembly
primary_assembly:ASM1334776v1:21:2382925:2383125:1[SNP_51788][**vipr1b**]
TGAAAACGTGTTTACTGTTAAACAATGATGAAACTTACTTCTGGCAGATGGCCGTACAGCAGCAGCAGCAGCAGCAGCATGAAGAACA
GCTGTGAGGCAT[T/C]CATCCTGTCTCCACTTTAGTTCTTATTCAGGACTCCGCAGTCCTGGCGACGCTCCTCCGCAAATCTCCCGAGGTG
AGTGTGTGTGTGTGTGTGAGAGAGA
>19 dna:primary_assembly
primary_assembly:ASM1334776v1:19:11066053:11066253:1[SNP_48228][**aqp8b**]
ATGAGAAGATGGAGATGGCAGAAGTGGAGACGTCTCTCGTGACTTCAGGGTCAAAACCAGCTCCGGCCAGACCTCCTAACAAATTCGA
GAAGTTATTTCA[G/T]CCCTGTCTGGGCGAGCTGGTGGGAACTACGTTCTTTGTCTTCATCGGGTGCGTGTCCGTCATAGAGAACGTGGA
GTCCACGGGGAGGCTTCAGCCGGCAC
