# Supplementary material for: Non-synonymous variation and protein structure of candidate genes associated with selection in farm and wild populations of turbot (Scophthalmus maximus)
Source: Sci Rep. 2023 Feb 21;13:3019. doi: 10.1038/s41598-023-29826-z (PMC9944912; doi:10.1038/s41598-023-29826-z)
Supplement: Supplementary file 5 — Supplementary Table S4. [file 41598_2023_29826_MOESM5_ESM.pdf]

Table S4: External (forward and reverse) and internal (extension) primers for the 18 genes including non-synonymous variants in *Scophthalmus maximus* genotyped with the MassARRAY technology

**Gene**	**Annotation**	**Extension**	**Forward**	**Reverse**
aqp8b	aquaporin 8b	ACAAATTCGAGAAGTTATTTCA	ACGTTGGATGTTCAGGGTCAAAACCAGCTC	ACGTTGGATGAAGAACGTAGTTCCCACCAG
cmtm3	CKLF-like MARVEL transmembrane domain containing 3	gtACCAAATTCAACGAGAGGTTTA	ACGTTGGATGTGCGCTCTTCTTGTTGTTTG	ACGTTGGATGTTGTCTCTCACCATGAGAGG
eya3	EYA transcriptional coactivator and phosphatase 3	GGCAAAGAAAGCCAAGC	ACGTTGGATGCGTTTTTGTGTGTATGCAGG	ACGTTGGATGCCAACCATGACTCTTTCTCC
hamp	hepcidin antimicrobial peptide	ttCAGCAGAAGCCACAGCCCTTGT	ACGTTGGATGTGCGGGTTATCCTCAGAACT	ACGTTGGATGAGAGCCACATCTCCCTTTGC
hgs	hepatocyte growth factor-regulated tyrosine kinase substrate	ccCAACAGCCGTACATGCCAGGCCAG	ACGTTGGATGTTTCAGCATTGCCAGGACAG	ACGTTGGATGCACAAACAACTGACCTGCTG
igf1rb	insulin-like growth factor 1b receptor	TTCAAAGACTCTAGTTTCTTC	ACGTTGGATGTAAAGGATGAACTGGAGCCG	ACGTTGGATGATTTTGTCCTGGTGGACCTG
fga-like	fibrinogen alpha chain-like transcript variant X2	tATAAGAGTTGGTTCGGAG	ACGTTGGATGCGCCGAGTACATGATAAGAG	ACGTTGGATGCGTGTACCCTGACACATGC
arhgap42	rho GTPase-activating protein 42 transcript variant X4	CAGCCTTTACACCCCGA	ACGTTGGATGAAGCCCCGCAGCCTTTACA	ACGTTGGATGGCCTTGGTACTCACTTTCTG
hmox.like	heme oxygenase-like	gTTTCTGATGTTTTAATTTCTTCAGG	ACGTTGGATGCGAGCATGCTTGAAGATGAG	ACGTTGGATGGAGTCTTCATCTCTGTCTCC
ciart	circadian-associated transcriptional repressor	gGATGTGTGGAGCCACTTCAGGTTA	ACGTTGGATGGAGCAGATGGGTGATGTGTG	ACGTTGGATGTGAGAGTCACCGACCTCACT
igfbp2	insulin-like growth factor-binding protein 2-B	CAAGAGGAGTTCAGAGAAATG	ACGTTGGATGCCCCAACTGTAACCAGTTTC	ACGTTGGATGTTTGCTGAGCTGTGCTAGAG
myb	v-myb avian myeloblastosis viral oncogene homolog transcript variant X1	aggGAGTTCTCCCGGCGAGGAGG	ACGTTGGATGAGCACGTACTTTCGCTCTCG	ACGTTGGATGGAGCCTGCTGCAGTTTAAAT
paxbp1	PAX3 and PAX7 binding protein 1	CCCGTTCATGGAGAATC	ACGTTGGATGGGAGGAAATCCCGTTCATGG	ACGTTGGATGCGTTGCTGTTGAAGTTGTCC
slc12a3	solute carrier family 12 member 3	CAACCCCAAGCAGGACTCTGATT	ACGTTGGATGACATGGTGAGTCGCTTCAGG	ACGTTGGATGCCTCCTCAGAGATCATCCAG
sstr3	somatostatin receptor 3 transcript variant X2	AGCTCCCGGCAGCTCTTGT	ACGTTGGATGAGCAGTTTCCAGCCGCAGAG	ACGTTGGATGGGCTTTGTCCTTGTTGTCCG
tshr	thyroid stimulating hormone receptor	gggCGAAACAGTCCCAGACC	ACGTTGGATGATAGCGTGGTCGAAACAGTC	ACGTTGGATGTCACCACTCCTCCAGTCTTG
vipr1b	vasoactive intestinal peptide receptor 1b	tgCTAAAGTGGAGACAGGATG	ACGTTGGATGACTGCGGAGTCCTGAATAAG	ACGTTGGATGCAGCATGAAGAACAGCTGTG
hbaD	hemoglobin subunit alpha-D	GCAGAGGAGCCAGGGTGA	ACGTTGGATGTGCATTGTTTGCAGAGGAGC	ACGTTGGATGAAGATTGTCCTGGCCATAGC
