# Supplementary material for: Non-synonymous variation and protein structure of candidate genes associated with selection in farm and wild populations of turbot (Scophthalmus maximus)
Source: Sci Rep. 2023 Feb 21;13:3019. doi: 10.1038/s41598-023-29826-z (PMC9944912; doi:10.1038/s41598-023-29826-z)
Supplement: Supplementary file 6 — Supplementary Table S5. [file 41598_2023_29826_MOESM6_ESM.pdf]

**Table S5**: Genotyping information of all individuals and genes analysed in turbot (*Scophthalmus maximus)*

| **Sample_Name** | ***fga-like*** | ***arhgap42*** | ***hmox*** | ***ciart*** | ***igfbp2*** | ***aqp8b*** | ***cmtm3*** | ***eya3*** | ***hamp*** | ***hgs*** | ***igf1rb*** | ***myb*** | ***paxbp1*** | ***slc12a3*** | ***sstr3*** | ***tshr*** | ***vipr1b*** | ***hbaD*** |
|---|---|---|---|---|---|---|---|---|---|---|---|---|---|---|---|---|---|---|
| Farm 1 | G | C | AG | A | G | G | TC | G | TA | C | GA | C | GA | G | C | AT | T | G |
| Farm 1 | GT | C | G | A | G | G | TC | GT | TA | C | G | C | A | G | C | T | TC | GA |
| Farm 1 | GT | C | G | GA | G | G | T | GT | TA | C | A | C | A | G | C | AT | C | G |
| Farm 1 | GT | C | AG | GA | GA | G | T | G | TA | C | A | C | GA | G | C | AT | T | G |
| Farm 1 | GT | C | G | A | G | G | TC | GT | TA | C | A | C | A | G | C | AT | C | GA |
| Farm 1 | GT | C | AG | G | G | GT | TC | T | TA | CA | A | C | A | G | C | T | TC | GA |
| Farm 1 | GT | C | G | A | G | G | T | GT | TA | C | A | C | A | CG | C | AT | C | GA |
| Farm 1 | GT | CA | G | GA | GA | G | T | G | TA | C | A | C | GA | G | C | AT | TC | GA |
| Farm 1 | GT | CA | G | A | G | G | T | G | A | C | GA | C | A | G | C | AT | C | G |
| Farm 1 | T | CA | G | GA | GA | G | T | G | TA | C | A | C | A | G | C | AT | C | GA |
| Farm 1 | GT | C | AG | G | G | G | TC | G | TA | C | A | C | A | G | C | T | T | G |
| Farm 1 | T | C | G | A | G | G | T | GT | T | C | GA | C | A | G | C | AT | TC | GA |
| Farm 1 | GT | C | G | A | GA | G | T | G | TA | C | GA | CT | A | G | C | AT | TC | G |
| Farm 1 | G | CA | AG | A | G | G | T | G | TA | C | GA | C | GA | CG | C | T | C | G |
| Farm 2 | G | CA | G | GA | G | G | T | G | A | C | A | C | A | CG | C | T | T | G |
| Farm 2 | GT | C | G | A | G | G | T | G | A | C | A | C | A | G | C | T | TC | G |
| Farm 2 | G | C | G | GA | G | G | T | G | TA | C | A | C | A | G | C | T | TC | G |
| Farm 2 | T | C | G | GA | G | G | T | T | TA | C | A | CT | A | CG | C | T | TC | G |
| Farm 2 | GT | C | G | GA | G | G | T | GT | A | C | A | C | A | G | C | T | C | G |
| Farm 2 | GT | C | G | GA | G | G | T | GT | TA | C | A | C | GA | G | C | T | C | G |
| Farm 2 | GT | C | G | A | G | G | T | GT | A | C | A | C | GA | G | C | AT | C | G |
| Farm 2 | GT | C | G | GA | G | G | T | T | A | C | A | CT | A | CG | C | T | T | G |
| Farm 2 | GT | C | G | A | G | G | T | GT | TA | C | A | C | A | G | C | T | C | G |
| Farm 2 | GT | C | G | GA | G | G | T | GT | A | C | GA | C | GA | G | C | T | C | G |

| | | | | | | | | | | | | | | | | | |
|---|---|---|---|---|---|---|---|---|---|---|---|---|---|---|---|---|---|
| Farm 2 | G | C | AG | GA | G | G | T | G | TA | C | GA | C | GA | G | C | A | T | G |
| BAS-N1 | GT | C | G | A | G | G | T | GT | TA | C | A | C | G | G | C | AT | C | G |
| BAS-N2 | G | C | G | A | G | G | T | GT | TA | C | A | C | A | G | C | A | C | G |
| BAS-N3 | T | C | G | A | G | G | T | GT | TA | C | A | C | GA | G | C | A | TC | G |
| BAS-N4 | GT | C | G | A | G | G | T | GT | T | C | A | C | A | G | C | A | C | G |
| BAS-N5 | G | C | G | A | G | G | T | G | A | C | A | C | A | CG | C | AT | C | G |
| BAS-N6 | T | C | G | A | G | G | T | GT | TA | C | A | C | GA | G | C | A | C | G |
| BAS-N7 | G | C | G | A | G | G | TC | G | A | C | A | C | GA | G | C | A | TC | G |
| BAS-N8 | GT | C | G | A | G | G | T | T | A | C | A | C | GA | G | C | AT | TC | G |
| BAS-N9 | GT | C | G | A | G | G | T | G | A | C | A | C | A | G | C | AT | C | G |
| BAS-N10 | GT | C | G | A | G | G | T | G | A | C | A | C | A | G | C | AT | C | G |
| BAS-N11 | G | C | G | A | G | G | T | GT | A | CA | A | C | GA | G | C | AT | C | G |
| BAS-N12 | G | C | G | GA | G | G | T | GT | A | C | A | C | A | G | C | AT | C | G |
| BAS-N13 | G | C | G | GA | G | G | T | T | A | C | A | C | A | G | C | A | TC | G |
| BAS-N14 | G | C | G | A | G | G | T | GT | A | C | A | C | GA | G | C | AT | C | G |
| BAS-N15 | GT | C | G | GA | G | G | T | GT | A | C | A | C | G | G | C | T | TC | G |
| BAS-N16 | G | C | G | A | G | G | T | T | T | C | A | C | A | G | C | T | C | G |
| BAS-N17 | G | CA | G | GA | G | G | T | GT | A | C | A | C | A | G | C | T | TC | G |
| BAS-N18 | GT | C | G | A | G | G | T | GT | TA | C | A | C | GA | G | C | A | C | G |
| BAS-N19 | G | C | G | GA | G | G | T | G | A | C | A | C | A | G | C | AT | C | G |
| BAS-N20 | G | C | G | A | G | G | TC | GT | A | C | A | C | A | G | C | T | C | G |
| BAS-N21 | G | C | G | A | G | G | TC | T | A | C | A | C | A | G | C | AT | TC | G |
| BAS-N22 | G | C | G | G | G | G | T | GT | A | C | A | C | A | G | C | AT | C | G |
| BAS-N23 | G | C | AG | G | G | G | T | T | TA | C | A | C | GA | G | C | AT | C | G |
| BAS-N24 | GT | C | G | A | G | G | T | G | TA | C | A | C | GA | G | C | T | TC | G |
| BAS-N25 | GT | C | G | GA | G | G | T | GT | A | C | A | C | GA | G | C | AT | C | G |
| NS-S1 | G | C | G | G | G | G | T | T | TA | C | A | C | A | G | C | AT | C | G |

| | | | | | | | | | | | | | | | | | |
|---|---|---|---|---|---|---|---|---|---|---|---|---|---|---|---|---|---|
| NS-S2 | T | C | G | GA | G | G | T | T | A | C | A | C | GA | CG | C | AT | C | GA |
| NS-S3 | T | C | AG | GA | G | G | T | G | A | C | A | C | A | CG | C | T | TC | G |
| NS-S4 | G | C | G | G | G | G | T | GT | A | C | A | C | A | G | C | AT | C | G |
| NS-S5 | GT | C | G | GA | G | G | T | GT | TA | C | A | C | G | G | C | T | TC | G |
| NS-S6 | G | C | G | G | G | G | T | GT | A | C | A | C | A | CG | C | T | TC | G |
| NS-S7 | G | C | G | GA | G | G | T | T | TA | C | A | C | A | CG | C | T | TC | G |
| NS-S8 | GT | C | G | GA | G | G | T | GT | A | C | GA | C | GA | G | C | T | C | G |
| NS-S9 | GT | C | G | A | G | G | TC | G | TA | C | GA | C | A | CG | C | T | C | G |
| NS-S10 | GT | C | AG | GA | G | G | TC | T | A | C | A | C | A | G | C | AT | C | G |
| NS-S11 | T | C | G | G | G | G | TC | GT | TA | C | A | CT | A | G | C | A | C | G |
| NS-S12 | G | C | G | GA | G | G | T | GT | TA | CA | A | C | G | G | C | T | T | G |
| NS-S13 | GT | C | G | G | G | G | TC | GT | A | CA | A | C | GA | G | C | AT | C | G |
| NS-S14 | G | C | G | GA | G | G | T | T | A | C | A | C | A | G | C | T | C | G |
| NS-S15 | GT | C | AG | G | G | G | T | T | A | C | A | C | GA | CG | C | T | TC | G |
| NS-S16 | GT | C | G | G | G | G | T | G | TA | C | GA | C | A | G | C | T | C | G |
| NS-S17 | GT | CA | G | G | G | G | T | G | A | C | A | C | G | G | C | T | T | G |
| NS-S18 | GT | C | AG | GA | G | G | T | G | TA | C | A | C | A | G | C | AT | TC | G |
| NS-S19 | GT | C | G | G | G | G | TC | GT | T | C | GA | C | GA | G | C | T | TC | G |
| NS-S20 | GT | C | G | GA | G | G | TC | T | A | C | GA | C | A | G | C | AT | TC | G |
| NS-S21 | G | CA | AG | A | G | G | T | G | A | C | A | C | GA | G | C | A | T | G |
| NS-S22 | T | CA | G | GA | G | G | T | GT | A | C | A | C | GA | CG | C | T | T | G |
| NS-S23 | GT | C | AG | GA | G | G | TC | T | TA | C | A | C | A | CG | C | T | T | G |
| NS-S24 | G | C | G | GA | G | G | TC | G | TA | CA | A | C | GA | G | C | A | TC | G |
| Farm 3 | GT | C | G | G | G | G | T | G | TA | C | GA | C | A | CG | C | T | TC | GA |
| Farm 3 | G | C | AG | GA | G | GT | TC | G | T | C | A | C | A | G | C | T | T | GA |
| Farm 3 | GT | C | G | G | G | GT | T | GT | TA | CA | A | C | A | G | C | AT | T | GA |
| Farm 3 | G | C | G | GA | G | G | TC | GT | A | C | GA | C | A | G | C | T | C | G |

| | | | | | | | | | | | | | | | | | | |
|---|---|---|---|---|---|---|---|---|---|---|---|---|---|---|---|---|---|---|
| Farm 3 | GT | C | G | G | G | GT | T | GT | TA | C | A | C | A | G | C | A | C | G |
| Farm 3 | GT | C | G | GA | G | G | T | G | TA | C | GA | C | GA | CG | C | T | T | G |
| Farm 3 | GT | C | G | GA | G | G | T | GT | TA | C | GA | C | A | G | C | AT | TC | G |
| Farm 3 | GT | C | G | G | G | G | T | GT | A | C | A | C | GA | G | C | AT | TC | GA |
| Farm 3 | G | C | AG | A | G | G | TC | GT | TA | C | A | C | GA | G | C | T | T | G |
| Farm 3 | G | CA | AG | GA | G | GT | TC | G | A | CA | A | C | GA | G | C | AT | C | G |
| Farm 3 | GT | C | G | A | GA | G | T | T | TA | C | A | C | A | CG | C | AT | T | GA |
| Farm 3 | T | C | G | G | G | G | TC | T | A | C | GA | C | A | CG | C | AT | T | GA |
| Farm 3 | GT | C | G | GA | G | G | T | GT | TA | C | GA | C | A | G | C | AT | TC | G |
| Farm 3 | GT | C | G | GA | G | G | TC | G | T | C | A | C | GA | G | C | T | TC | G |
| Farm 3 | T | C | G | G | G | G | T | G | A | C | A | C | G | CG | C | AT | TC | G |
| Farm 3 | T | CA | G | G | G | G | T | GT | TA | C | GA | C | GA | CG | C | T | T | G |
| Farm 3 | GT | C | G | GA | G | G | T | T | A | CA | A | C | GA | CG | C | AT | TC | GA |
| Farm 3 | G | C | G | G | GA | G | T | T | A | C | GA | C | A | CG | C | T | TC | G |
| Farm 3 | GT | CA | G | A | G | GT | T | GT | TA | CA | GA | C | A | G | C | AT | C | GA |
| Farm 3 | GT | C | AG | GA | G | G | T | G | TA | CA | GA | C | A | CG | C | AT | T | GA |
| Farm 3 | GT | CA | G | A | G | G | TC | GT | TA | C | G | C | A | G | C | A | C | GA |
| Farm 3 | GT | C | G | A | G | G | TC | G | TA | C | A | C | A | CG | C | AT | C | GA |
| Farm 3 | G | C | AG | GA | G | G | T | T | A | C | A | C | A | CG | C | A | C | GA |
| Farm 3 | GT | C | G | GA | GA | G | T | GT | A | C | A | C | GA | G | C | T | T | G |
| Farm 3 | T | C | G | G | G | GT | TC | G | TA | CA | A | C | A | G | C | AT | TC | G |

| | | | | | | | | | | | | | | | | | | |
|---|---|---|---|---|---|---|---|---|---|---|---|---|---|---|---|---|---|---|
| SP-W1 | GT | C | G | G | G | G | T | G | A | C | A | C | GA | G | C | A | TC | G |
| SP-W2 | GT | C | G | G | G | G | T | GT | A | C | A | C | G | CG | C | A | TC | G |
| SP-W3 | GT | C | AG | G | G | G | T | T | TA | C | A | CT | A | G | C | A | C | G |
| SP-W4 | T | C | G | G | G | G | T | T | TA | C | A | C | GA | G | C | AT | TC | G |
| SP-W5 | T | C | G | G | G | G | T | T | TA | C | A | C | GA | G | C | AT | TC | G |

| | | | | | | | | | | | | | | | | | | |
|---|---|---|---|---|---|---|---|---|---|---|---|---|---|---|---|---|---|---|
| SP-W6 | GT | C | G | G | G | G | T | G | A | C | A | CT | A | G | C | AT | C | G |
| SP-W7 | GT | C | G | GA | G | G | T | GT | TA | C | A | C | A | G | C | T | C | G |
| SP-W8 | G | C | AG | GA | G | G | T | T | A | C | A | C | GA | G | C | T | C | G |
| SP-W9 | GT | C | AG | G | G | G | T | T | A | C | A | C | A | CG | C | T | TC | G |
| SP-W10 | G | C | AG | GA | G | G | TC | GT | A | C | GA | C | A | G | C | T | C | G |
| SP-W11 | G | C | G | GA | G | G | T | GT | T | C | A | C | A | G | C | AT | C | G |
| SP-W12 | T | C | AG | GA | G | G | TC | T | A | C | A | C | A | G | C | A | TC | G |
| SP-W13 | G | C | G | GA | G | G | T | G | TA | C | A | C | GA | CG | C | AT | C | G |
| SP-W14 | GT | C | G | A | G | GT | TC | T | A | C | A | C | A | G | C | AT | TC | G |
| SP-W15 | GT | C | AG | GA | G | G | T | T | A | C | A | C | A | G | C | T | C | GA |
| SP-W16 | GT | C | G | G | G | G | T | G | T | C | A | C | A | G | C | T | TC | G |
| SP-W17 | G | C | G | GA | G | G | T | G | A | C | A | C | A | C | C | T | C | G |
| SP-W18 | G | C | AG | GA | G | G | T | GT | A | C | A | C | GA | CG | C | A | T | G |
| SP-W19 | G | C | AG | GA | G | G | T | GT | A | C | A | C | GA | CG | C | A | TC | G |
| SP-W20 | GT | C | G | GA | G | G | T | T | A | C | A | C | A | G | C | T | C | G |
| SP-W21 | T | C | G | G | G | G | T | T | A | C | A | C | A | CG | C | AT | TC | G |
| SP-W22 | GT | C | G | GA | G | G | T | GT | A | C | A | C | GA | CG | C | T | C | G |
| SP-W23 | T | C | G | GA | G | G | T | GT | A | C | A | C | A | G | C | T | C | G |
| SP-W24 | GT | C | G | GA | G | G | T | GT | TA | C | A | C | GA | G | C | AT | C | G |
| SP-W25 | GT | C | G | GA | G | G | T | T | TA | C | A | C | G | G | C | T | TC | G |
| SP-W26 | G | C | G | GA | G | G | T | GT | TA | C | A | C | A | G | C | T | C | G |
| BB-SE1 | G | C | G | GA | G | G | TC | T | A | C | GA | C | GA | CG | C | A | No Call | G |
| BB-SE2 | G | C | AG | GA | G | G | T | GT | A | CA | GA | C | A | G | C | T | TC | G |
| BB-SE3 | GT | C | G | G | G | G | T | T | TA | C | A | C | A | G | C | T | T | G |
| BB-SE4 | GT | C | AG | A | G | G | T | GT | A | C | A | C | A | C | C | AT | TC | G |
| BB-SE5 | GT | C | G | G | G | G | TC | G | A | C | A | C | A | G | C | AT | T | G |
| BB-SE6 | GT | C | AG | G | G | G | T | T | A | C | A | C | A | G | C | T | C | G |

| | | | | | | | | | | | | | | | | | |
|---|---|---|---|---|---|---|---|---|---|---|---|---|---|---|---|---|---|
| BB-SE7 | GT | C | G | G | G | G | T | T | TA | C | A | C | A | G | C | T | TC | G |
| BB-SE8 | G | C | G | GA | G | G | T | GT | TA | C | GA | C | GA | CG | C | T | TC | G |
| BB-SE9 | GT | C | G | G | G | G | T | G | TA | C | GA | C | A | CG | C | A | T | G |
| BB-SE10 | GT | C | G | G | GA | G | T | GT | TA | C | A | C | A | G | C | AT | T | G |
| BB-SE11 | GT | CA | G | A | G | G | T | G | A | C | A | C | A | G | C | AT | C | G |
| BB-SE12 | GT | C | G | G | G | G | T | G | TA | C | A | C | GA | G | C | T | TC | G |
| BB-SE13 | GT | C | G | G | G | G | TC | G | A | C | A | CT | A | G | C | T | TC | GA |
| BB-SE14 | GT | C | G | GA | G | G | T | GT | TA | C | A | C | A | G | C | AT | C | G |
| BB-SE15 | G | C | AG | A | G | G | T | GT | TA | C | A | C | GA | G | C | AT | TC | G |
| BB-SE16 | GT | C | AG | GA | G | G | T | GT | A | C | GA | C | G | G | C | AT | C | G |
| BB-SE17 | T | C | G | GA | G | G | T | GT | TA | C | GA | C | GA | G | C | T | TC | G |
| BB-SE18 | T | C | AG | A | G | G | T | T | TA | C | A | C | A | G | C | AT | TC | G |
| BB-SE19 | GT | C | G | GA | G | G | T | G | A | C | A | C | A | G | C | T | T | G |
| BB-SE20 | T | C | G | G | G | G | T | GT | TA | C | A | C | GA | G | C | T | C | G |
| BB-SE21 | G | C | G | G | G | G | T | G | TA | C | A | C | A | CG | C | T | TC | G |
| BB-SE22 | GT | C | AG | G | G | G | T | T | A | C | A | C | GA | G | C | T | T | G |
| BB-SE23 | GT | C | G | G | G | G | T | GT | A | C | A | C | GA | G | C | T | TC | G |
| BB-SE24 | GT | C | G | G | G | G | T | G | A | C | A | C | GA | G | C | T | TC | G |
| BB-SE25 | GT | C | AG | GA | G | G | T | G | TA | C | A | C | A | G | C | T | TC | G |
| BLS-N1 | G | C | G | G | G | G | T | T | A | C | A | C | A | G | C | T | TC | G |
| BLS-N2 | GT | C | AG | G | G | G | T | T | TA | C | A | C | GA | C | C | AT | T | G |
| BLS-N3 | G | C | G | GA | G | G | TC | T | A | C | A | C | A | G | C | T | TC | G |
| BLS-N4 | G | C | AG | GA | G | G | T | T | T | C | A | C | A | G | C | AT | TC | G |
| BLS-N5 | G | C | G | G | G | G | T | T | TA | C | A | C | A | CG | C | AT | TC | G |
| BLS-N6 | G | C | G | GA | G | G | T | T | T | C | A | C | GA | G | C | AT | TC | G |
| BLS-N7 | G | C | G | A | G | G | T | T | TA | C | A | C | A | CG | C | T | TC | G |
| BLS-N8 | GT | C | G | G | G | G | T | T | A | C | A | C | A | C | C | AT | TC | G |

| | | | | | | | | | | | | | | | | | |
|---|---|---|---|---|---|---|---|---|---|---|---|---|---|---|---|---|---|
| BLS-N9 | G | C | AG | GA | G | G | T | T | TA | C | A | C | A | CG | C | T | T | G |
| BLS-N10 | GT | C | G | GA | G | G | T | T | TA | C | A | C | GA | CG | C | T | TC | G |
| BLS-N11 | GT | C | G | GA | G | G | T | T | TA | C | A | C | GA | CG | C | T | TC | G |
| BLS-N12 | G | C | G | GA | G | G | T | T | A | C | A | C | A | G | C | T | TC | G |
| BLS-N13 | G | C | G | GA | G | G | T | T | T | C | A | C | GA | CG | C | AT | C | G |
| BLS-N14 | G | C | G | GA | G | G | T | T | A | C | A | C | A | G | C | AT | C | G |
| BLS-N15 | G | C | AG | GA | G | G | T | T | A | C | A | C | A | G | C | T | TC | G |
| BLS-N16 | G | C | G | A | G | G | T | T | T | C | A | C | A | CG | C | T | T | G |
| BLS-N17 | GT | C | AG | G | G | G | T | T | T | C | A | CT | A | G | C | T | TC | G |
| NOR1 | GT | C | G | GA | G | G | T | T | A | C | A | C | A | G | C | AT | C | G |
| NOR2 | GT | C | G | G | G | G | T | G | TA | C | A | C | A | G | C | AT | TC | G |
| NOR3 | GT | C | AG | GA | G | G | T | T | A | C | A | C | A | G | C | AT | C | G |
| NOR4 | T | C | G | GA | G | G | T | GT | TA | C | A | C | GA | C | C | T | C | G |
| NOR5 | G | C | AG | GA | G | G | T | GT | TA | C | GA | C | GA | G | C | T | T | G |
| NOR6 | G | C | G | GA | G | G | TC | GT | A | C | A | C | A | G | C | T | TC | GA |
| NOR7 | G | C | G | A | G | G | T | G | A | C | A | C | A | G | C | AT | TC | G |
| NOR8 | T | C | G | G | G | G | T | G | A | C | A | C | G | CG | C | T | T | G |
| NOR9 | T | C | G | GA | G | G | TC | GT | A | C | GA | C | GA | G | C | AT | C | G |
| NOR10 | G | C | G | GA | G | G | T | GT | TA | C | GA | C | GA | G | C | AT | TC | G |
| NOR11 | G | CA | G | G | G | G | T | T | TA | C | A | C | GA | G | C | AT | C | G |
| NOR12 | GT | C | G | GA | G | G | T | GT | TA | C | A | C | G | CG | C | AT | TC | G |
| NOR13 | GT | CA | G | A | G | G | T | T | A | C | A | C | A | C | C | AT | TC | G |
| NOR14 | GT | C | G | G | G | G | TC | T | TA | C | A | C | A | G | C | AT | TC | G |
| NOR15 | GT | C | G | G | G | G | T | GT | A | C | A | C | A | G | C | T | T | G |
| NOR16 | G | C | AG | G | G | G | T | GT | TA | C | A | C | GA | CG | C | AT | C | G |
| NOR17 | GT | C | G | GA | G | G | T | T | A | C | A | C | GA | G | C | AT | C | G |
| NOR18 | GT | C | G | GA | G | G | T | T | A | C | A | C | GA | G | C | AT | C | G |

| NOR19 | GT | C | G | GA | G | G | T | T | TA | CA | A | C | GA | G | C | T | T | G |
|---|---|---|---|---|---|---|---|---|---|---|---|---|---|---|---|---|---|---|
| T1 | GT | C | G | GA | G | G | T | G | TA | C | GA | C | A | G | C | AT | T | G |
| T2 | T | C | AG | G | G | G | T | T | A | C | A | C | GA | G | C | A | TC | G |
| T3 | G | C | AG | GA | G | G | TC | G | A | C | A | C | A | G | C | AT | T | G |
| T4 | GT | C | AG | A | G | G | T | GT | TA | C | GA | C | A | CG | C | AT | TC | G |
| T5 | GT | C | G | G | G | G | T | GT | A | C | GA | C | A | G | C | AT | C | G |
| T6 | G | C | G | A | G | G | T | T | TA | C | A | C | A | G | C | T | C | G |
| T7 | GT | C | G | G | G | G | T | T | TA | C | A | C | A | G | C | AT | TC | G |
| T8 | G | C | G | GA | G | G | T | G | A | C | A | C | A | CG | C | T | C | G |
| T9 | G | C | G | GA | G | G | T | G | T | C | GA | C | A | G | C | T | TC | G |
| T10 | GT | C | G | A | G | G | T | G | A | C | A | C | A | G | C | T | TC | G |
| T11 | GT | C | G | A | G | G | TC | G | A | C | A | C | GA | G | C | A | C | G |
| T12 | G | CA | G | GA | G | G | T | GT | A | C | A | C | GA | G | C | T | TC | G |
| T13 | GT | C | G | A | G | G | T | GT | A | C | GA | C | GA | G | C | A | C | G |
| T14 | T | C | G | GA | G | G | T | GT | A | C | A | C | GA | G | C | AT | TC | G |
| T15 | G | C | AG | GA | G | G | T | GT | T | C | GA | C | A | CG | C | AT | C | G |
| T16 | GT | CA | G | G | G | G | T | G | A | C | A | C | GA | G | C | T | C | G |
| T17 | T | C | G | GA | G | G | T | GT | A | C | A | C | A | G | C | AT | C | GA |
| T18 | G | C | AG | GA | G | G | T | GT | A | C | A | CT | GA | CG | C | AT | TC | G |
| T19 | GT | C | G | A | G | G | T | T | TA | C | A | CT | GA | G | C | AT | TC | G |
| T20 | GT | C | G | G | G | G | T | GT | TA | C | A | C | GA | CG | C | AT | T | G |
| T21 | G | C | G | G | G | G | T | G | A | C | A | C | A | CG | C | AT | C | G |
| T22 | G | C | G | G | G | G | TC | T | TA | C | A | C | GA | G | C | AT | T | G |
| T23 | G | C | G | GA | G | G | T | T | TA | C | A | C | GA | G | C | T | TC | G |
| T24 | G | C | G | GA | G | G | T | T | A | C | A | C | GA | G | C | T | TC | G |
| T25 | T | C | AG | A | G | G | T | G | T | C | A | C | GA | G | C | AT | T | G |
| BLS-S1 | G | C | G | G | G | G | T | T | A | C | A | C | A | CG | C | T | TC | G |

| | | | | | | | | | | | | | | | | | | |
|---|---|---|---|---|---|---|---|---|---|---|---|---|---|---|---|---|---|---|
| BLS-S2 | GT | C | G | GA | G | G | T | T | TA | C | A | C | A | G | C | T | C | G |
| BLS-S3 | G | C | G | A | G | G | T | T | T | C | A | C | G | CG | C | T | T | G |
| BLS-S4 | GT | C | AG | G | G | G | T | T | TA | C | A | C | A | CG | C | T | TC | G |
| BLS-S5 | G | C | AG | GA | G | G | T | T | A | C | A | C | A | G | C | T | TC | G |
| BLS-S6 | G | C | G | G | G | G | T | T | TA | C | A | C | A | G | C | T | TC | G |
| BLS-S7 | G | C | G | G | G | G | T | T | A | C | A | C | GA | CG | C | A | C | G |
| BLS-S8 | G | C | G | G | G | G | T | T | T | C | A | CT | GA | C | C | T | C | G |
| BLS-S9 | G | C | G | G | G | G | T | T | TA | C | A | C | A | CG | C | T | C | G |
| BLS-S10 | GT | C | G | G | G | G | T | T | TA | C | A | C | A | G | C | T | C | G |
| BLS-S11 | G | C | A | GA | G | G | T | T | A | C | A | C | A | G | C | T | C | G |
| BLS-S12 | G | C | G | G | G | G | T | T | TA | C | A | C | A | CG | C | AT | TC | G |
| BLS-S13 | G | C | AG | GA | G | G | T | T | TA | C | A | C | GA | G | C | T | C | G |
| BLS-S14 | G | C | AG | GA | G | G | T | T | TA | C | A | C | GA | CG | C | T | T | G |
| BLS-S15 | G | C | AG | G | G | G | TC | GT | TA | C | A | C | GA | CG | C | T | T | G |
| BLS-S16 | G | C | AG | G | G | G | T | GT | A | C | A | CT | A | G | C | AT | T | G |
| BLS-S17 | G | C | G | GA | G | G | T | T | A | C | A | C | A | G | C | AT | C | G |
| BLS-S18 | G | C | A | A | G | G | T | T | T | C | A | C | A | CG | C | AT | C | G |
| BLS-S19 | G | C | AG | GA | G | G | T | T | T | C | A | C | GA | C | C | T | C | G |
| BLS-S20 | G | C | AG | G | G | G | T | T | T | C | A | C | A | G | C | AT | C | G |
| BLS-S21 | G | C | G | GA | G | G | T | T | T | C | A | C | A | CG | C | T | T | G |
| BLS-S22 | G | C | AG | A | G | G | TC | T | T | C | A | C | A | CG | C | AT | TC | G |
| BLS-S23 | G | C | G | A | G | G | T | T | TA | C | A | C | A | CG | C | T | TC | G |
| BLS-S24 | G | C | G | G | G | G | T | T | TA | C | A | CT | G | C | C | AT | TC | G |
| BLS-S25 | G | C | G | G | G | G | T | T | TA | C | A | CT | G | C | C | AT | TC | G |
| BLS-S26 | G | C | G | GA | G | G | T | GT | TA | C | A | CT | A | CG | C | AT | T | G |
| BLS-S27 | G | C | G | A | G | G | TC | T | TA | C | A | C | A | G | C | T | TC | G |
| BLS-S28 | G | C | G | GA | G | G | TC | T | A | C | A | C | A | CG | C | T | TC | G |

| | | | | | | | | | | | | | | | | | | |
|---|---|---|---|---|---|---|---|---|---|---|---|---|---|---|---|---|---|---|
| AD1 | G | C | AG | GA | G | G | T | GT | A | C | A | C | A | G | C | A | T | G |
| AD2 | G | C | G | GA | G | G | T | G | TA | C | A | C | A | G | C | A | TC | G |
| AD3 | G | C | AG | G | G | G | TC | GT | A | C | A | C | A | C | C | A | TC | G |
| AD4 | G | C | G | GA | G | G | T | GT | A | C | A | C | A | G | C | A | TC | G |
| AD5 | G | C | AG | GA | G | G | TC | GT | A | C | A | C | GA | G | C | A | TC | G |
| AD6 | G | C | AG | G | G | G | T | T | A | C | A | C | GA | CG | C | A | C | G |
| AD7 | GT | C | G | G | G | G | T | GT | A | C | A | C | GA | C | C | A | TC | G |
| AD8 | GT | C | G | G | G | G | T | GT | A | C | A | C | GA | CG | C | A | TC | G |
| AD9 | G | C | G | GA | G | G | TC | T | A | C | A | C | A | G | C | A | TC | G |
| AD10 | G | C | G | G | G | G | T | GT | A | C | A | C | A | C | C | AT | TC | G |
| AD11 | G | C | G | GA | G | G | T | G | A | C | A | C | GA | G | C | A | TC | G |
| AD12 | G | C | G | G | G | G | C | T | TA | C | A | C | GA | G | C | A | T | G |
| AD13 | G | C | G | GA | G | G | T | G | T | C | A | C | A | C | C | AT | TC | G |
| AD14 | G | C | AG | G | G | G | T | GT | A | C | A | C | GA | CG | C | AT | C | G |
| AD15 | G | C | AG | GA | G | G | T | G | TA | C | A | C | GA | CG | C | A | TC | G |
| AD16 | GT | C | G | G | G | G | T | G | TA | C | A | C | G | G | C | A | T | G |
| AD17 | GT | C | G | GA | G | G | T | GT | A | C | A | C | GA | CG | C | AT | TC | G |
| AD18 | GT | C | AG | GA | G | G | T | G | A | C | A | C | A | CG | C | AT | TC | G |
| AD19 | G | C | A | G | G | G | T | T | A | C | A | C | A | CG | C | A | TC | G |
| AD20 | G | C | G | G | G | G | T | T | A | C | A | C | A | G | C | A | TC | G |
| AD21 | G | C | AG | A | G | G | C | GT | A | C | A | C | GA | CG | C | A | TC | G |
| AD22 | GT | C | AG | G | G | G | TC | GT | TA | C | A | C | A | G | C | A | TC | G |
| AD23 | G | C | AG | GA | G | G | T | G | A | C | A | C | GA | C | C | A | C | G |
| AD24 | G | C | AG | GA | G | G | TC | T | TA | C | A | C | GA | G | C | A | T | G |
| AD25 | G | C | G | G | G | G | TC | T | TA | C | A | C | GA | G | C | A | TC | G |
| BB-FR1 | GT | C | G | G | G | G | T | T | A | C | A | C | GA | CG | C | AT | C | G |
| BB-FR2 | G | C | G | G | G | G | T | GT | A | C | A | C | A | C | C | T | TC | G |

| | | | | | | | | | | | | | | | | | | |
|---|---|---|---|---|---|---|---|---|---|---|---|---|---|---|---|---|---|---|
| BB-FR3 | GT | C | G | A | G | G | T | GT | TA | C | A | C | A | G | C | T | TC | G |
| BB-FR4 | GT | CA | G | G | G | G | T | T | A | C | A | C | GA | G | C | T | T | G |
| BB-FR5 | G | C | G | GA | G | G | T | GT | A | C | A | C | A | G | C | AT | TC | G |
| BB-FR6 | G | C | G | GA | G | G | TC | GT | A | C | A | CT | GA | CG | C | T | T | G |
| BB-FR7 | G | C | G | GA | G | G | T | T | A | C | A | C | A | G | C | AT | C | G |
| BB-FR8 | GT | C | G | G | G | G | T | T | TA | C | A | C | A | G | C | A | C | G |
| BB-FR9 | G | C | G | GA | G | G | T | G | TA | C | A | CT | GA | G | C | AT | TC | G |
| BB-FR10 | GT | C | G | GA | G | G | T | GT | A | C | A | C | GA | CG | C | AT | T | G |
| BB-FR11 | GT | C | G | GA | G | G | T | G | A | C | A | C | A | G | C | T | C | G |
| BB-FR12 | G | C | G | GA | G | G | T | GT | TA | CA | A | C | GA | G | C | T | T | G |
| BB-FR13 | GT | C | G | GA | G | G | T | T | A | C | GA | CT | G | G | C | AT | TC | G |
| BB-FR14 | GT | C | G | GA | G | G | TC | GT | A | C | A | C | A | G | C | T | TC | G |
| BB-FR15 | GT | C | G | GA | G | G | TC | GT | A | C | A | C | A | G | C | T | TC | G |
| BB-FR16 | G | C | G | GA | G | G | T | GT | T | C | A | C | A | G | C | T | C | G |
| BB-FR17 | GT | C | G | G | G | G | T | GT | A | C | A | C | A | G | C | AT | T | G |
| BB-FR18 | T | C | AG | G | G | G | T | G | A | C | A | C | GA | G | C | A | TC | G |
| BB-FR19 | T | C | G | GA | G | G | TC | GT | A | C | A | C | GA | G | C | A | C | GA |
| BB-FR20 | T | C | AG | GA | G | G | T | T | TA | C | A | C | GA | G | C | T | T | G |
| BB-FR21 | G | C | G | GA | G | G | T | GT | A | C | A | CT | A | G | C | AT | TC | G |
| BB-FR22 | GT | C | G | GA | G | G | T | GT | A | C | A | CT | A | G | C | T | C | G |
| BB-FR23 | T | C | G | GA | G | G | T | GT | A | C | GA | C | A | G | C | T | TC | G |
| IR-E1 | G | C | G | GA | G | G | T | T | TA | C | A | C | A | G | C | T | T | G |
| IR-E2 | T | C | G | G | G | G | T | GT | TA | C | A | C | GA | G | C | AT | TC | G |
| IR-E3 | GT | C | G | GA | G | G | T | T | TA | C | A | C | GA | G | C | T | TC | G |
| IR-E4 | GT | C | G | G | G | G | TC | GT | TA | C | A | C | A | CG | C | A | TC | GA |
| IR-E5 | GT | C | G | GA | G | G | T | GT | TA | C | A | C | GA | G | C | T | C | G |
| IR-E6 | G | C | G | G | G | G | TC | T | A | CA | A | C | A | G | C | AT | TC | G |

| | | | | | | | | | | | | | | | | | | |
|---|---|---|---|---|---|---|---|---|---|---|---|---|---|---|---|---|---|---|
| IR-E7 | GT | C | G | GA | G | G | T | GT | T | C | A | C | A | G | C | AT | C | G |
| IR-E8 | T | C | A | GA | G | G | T | GT | TA | C | A | CT | GA | G | C | A | TC | G |
| IR-E9 | GT | C | G | GA | G | G | T | T | A | C | A | C | A | C | C | T | C | G |
| IR-E10 | GT | C | AG | GA | G | G | T | GT | A | C | GA | CT | GA | G | C | T | TC | G |
| IR-E11 | T | C | G | GA | G | G | T | GT | A | C | A | C | GA | G | C | T | TC | G |
| IR-E12 | GT | C | G | A | G | G | T | T | TA | C | A | C | GA | G | C | T | TC | G |
| IR-E13 | GT | C | A | GA | G | G | TC | G | TA | C | A | C | G | G | C | A | TC | G |
| IR-E14 | GT | C | G | GA | G | G | T | GT | A | C | GA | C | A | CG | C | T | TC | G |
| IR-E15 | GT | CA | G | GA | G | G | T | GT | A | C | A | C | A | CG | C | T | TC | G |
| IR-E16 | GT | C | G | GA | G | G | T | T | A | C | A | C | GA | CG | C | T | TC | G |
| IR-E17 | GT | C | G | G | G | G | T | G | A | C | GA | C | A | G | C | T | TC | G |
| IR-E18 | GT | C | G | G | G | G | TC | T | TA | C | GA | C | A | CG | C | T | C | G |
| IR-E19 | GT | C | G | G | G | G | T | GT | TA | C | A | C | GA | CG | C | AT | C | G |
| IR-E20 | G | C | G | G | G | G | T | G | TA | C | A | C | A | G | C | T | T | G |
| IR-E21 | G | C | AG | A | G | G | T | T | A | C | A | C | A | G | C | AT | C | G |
| IR-E22 | G | C | G | A | G | G | T | G | A | C | A | C | A | G | C | T | C | G |
| IR-E23 | G | CA | AG | GA | G | G | T | T | A | C | A | CT | A | G | C | AT | TC | GA |
| IR-E24 | G | C | G | A | G | G | T | GT | A | C | A | C | GA | G | C | T | TC | G |
| IR-E25 | GT | C | G | GA | G | G | T | GT | TA | C | A | C | A | G | C | T | TC | G |
| IR-W1 | G | C | AG | GA | G | G | T | G | A | C | A | C | A | G | C | T | TC | G |
| IR-W2 | G | C | A | GA | G | G | TC | G | TA | C | A | C | GA | G | C | T | C | G |
| IR-W3 | T | C | G | GA | G | G | TC | GT | A | C | A | C | G | CG | C | AT | C | G |
| IR-W4 | GT | C | G | A | G | G | C | G | T | C | A | C | GA | CG | C | T | TC | GA |
| IR-W5 | G | C | G | GA | G | G | T | GT | TA | C | A | C | A | G | C | T | T | G |
| IR-W6 | T | CA | G | GA | G | G | T | GT | TA | C | A | C | A | G | C | AT | C | G |
| IR-W7 | GT | C | AG | GA | G | G | T | GT | A | C | A | C | A | C | C | T | TC | G |
| IR-W8 | T | CA | G | GA | G | G | T | T | TA | C | A | C | A | G | C | AT | TC | GA |

| | | | | | | | | | | | | | | | | | | |
|---|---|---|---|---|---|---|---|---|---|---|---|---|---|---|---|---|---|---|
| IR-W9 | GT | C | G | G | G | G | TC | G | A | C | A | C | A | CG | C | T | C | G |
| IR-W10 | GT | C | G | A | G | G | T | GT | TA | C | A | C | A | CG | C | T | TC | G |
| IR-W11 | GT | C | G | GA | G | G | TC | T | A | C | A | C | A | G | C | T | TC | G |
| IR-W12 | T | C | AG | A | G | G | T | GT | A | C | A | CT | A | G | C | A | TC | G |
| IR-W13 | G | C | G | GA | G | G | T | GT | A | C | A | C | GA | G | C | AT | TC | G |
| IR-W14 | G | C | G | G | G | G | T | GT | A | C | GA | C | A | G | C | A | C | G |
| IR-W15 | GT | C | G | GA | G | G | T | GT | A | C | A | C | A | G | C | AT | T | G |
| IR-W16 | GT | C | G | GA | G | G | T | G | A | CA | A | C | A | G | C | AT | C | G |
| IR-W17 | T | C | G | A | G | G | T | GT | T | C | A | C | GA | G | C | A | TC | G |
| IR-W18 | GT | C | G | G | G | G | TC | GT | TA | C | A | C | A | G | C | T | TC | G |
| IR-W19 | G | C | G | A | G | G | T | G | TA | C | A | C | GA | G | C | AT | T | G |
| IR-W20 | G | C | G | G | G | G | T | GT | A | C | A | C | A | G | C | A | C | G |
| IR-W21 | G | C | G | G | G | G | T | GT | A | C | A | C | A | G | CT | T | TC | G |
| IR-W22 | T | C | AG | A | G | G | T | GT | A | C | A | CT | A | CG | C | AT | TC | G |
| IR-W23 | G | C | G | A | G | G | T | T | TA | C | A | C | GA | G | C | AT | TC | G |
| IR-W24 | GT | A | G | GA | G | G | T | GT | T | C | A | C | GA | G | C | AT | C | G |
| IR-W25 | GT | C | AG | G | G | G | T | GT | TA | C | A | C | A | CG | C | AT | C | G |
| ECH1 | GT | C | AG | GA | G | G | T | T | A | C | A | C | A | G | C | A | C | G |
| ECH2 | GT | C | G | G | G | G | T | G | TA | C | A | C | A | G | C | AT | TC | G |
| ECH3 | G | C | G | GA | G | G | T | G | A | C | A | C | A | G | C | A | C | G |
| ECH4 | GT | C | G | GA | G | G | T | T | TA | C | A | C | A | G | C | AT | TC | G |
| ECH5 | GT | C | AG | G | G | G | T | GT | T | C | A | C | A | G | C | AT | C | G |
| ECH6 | G | C | G | GA | G | G | C | G | A | C | G | C | A | G | C | T | C | G |
| ECH7 | GT | C | AG | GA | G | G | T | GT | A | C | GA | C | A | G | C | A | T | G |
| ECH8 | GT | C | G | G | G | G | TC | GT | T | C | A | C | A | G | C | AT | C | G |
| ECH9 | T | CA | AG | GA | G | G | T | GT | TA | C | A | C | GA | G | C | AT | TC | G |
| ECH10 | GT | C | A | A | G | G | T | GT | TA | C | A | C | GA | G | C | AT | C | G |

| | | | | | | | | | | | | | | | | | | |
|---|---|---|---|---|---|---|---|---|---|---|---|---|---|---|---|---|---|---|
| ECH11 | G | C | G | GA | G | G | T | G | A | C | GA | C | A | G | C | T | TC | G |
| ECH12 | G | C | G | GA | G | G | T | G | A | CA | A | C | A | CG | C | A | T | G |
| ECH13 | G | C | AG | GA | G | G | T | GT | TA | C | A | C | A | CG | C | A | TC | G |
| ECH14 | G | C | G | A | G | G | T | GT | A | C | A | C | A | G | C | T | T | G |
| ECH15 | G | C | G | GA | G | G | T | GT | A | C | A | C | GA | G | C | T | TC | G |
| ECH16 | GT | C | A | GA | G | G | T | GT | A | C | A | C | GA | G | C | A | TC | G |
| ECH17 | GT | CA | G | A | G | G | T | GT | A | C | A | C | GA | G | C | T | TC | G |
| ECH18 | T | C | G | G | G | G | T | G | TA | C | A | C | GA | G | C | T | TC | G |
