# Supplementary material for: Non-synonymous variation and protein structure of candidate genes associated with selection in farm and wild populations of turbot (Scophthalmus maximus)
Source: Sci Rep. 2023 Feb 21;13:3019. doi: 10.1038/s41598-023-29826-z (PMC9944912; doi:10.1038/s41598-023-29826-z)
Supplement: Supplementary file 7 — Supplementary Table S6. [file 41598_2023_29826_MOESM7_ESM.pdf]

Table S6. Stereochemical quality assessment by PROCHECK of the RoseTTAfold and I-TASSER models of turbot TSHR, PAXBP1, EYA3 and IGFBP2.

**Protein**	**Algorithm**				
		Core	Allowed	General	Disallowed
TSHR	RoseTTAfold	85.4%	13.1%	0.9%	0.6%
	I-TASSER	63.7%	27.5%	5.5%	3.3%
PAXB1	RoseTTAfold	90.2%	8.0%	1.0%	0.9%
	I-TASSER	71.8%	21.6%	4.0%	2.6%
EYA3	RoseTTAfold	90.0%	8.7%	1.0%	0.4%
	I-TASSER	53.6%	38.1%	4.3%	3.9%
IGFBP2	RoseTTAfold	92.5%	6.6%	0.9%	0.0%
	I-TASSER	43.8%	42.0%	9.3%	4.9%
